# Humanoid Robotic Loading Enhances Mechanotransduction in Tendon Tissue Engineering

**DOI:** 10.34133/cbsystems.0542

**Published:** 2026-03-24

**Authors:** Zekun Liu, Jinrong Lin, Tania Choreno Machain, Muhammad Hanif Nadhif, Yuyang Wei, Nicole Dvorak, Dylan Yeo, Yu Kiu Victor Chan, Alona Kharchenko, Rafael Hostettler, Antoine Jerusalem, Sarah Waters, Sarah Snelling, Pierre-Alexis Mouthuy

**Affiliations:** ^1^ Botnar Institute of Musculoskeletal Sciences, Nuffield Department of Orthopaedics, Rheumatology and Musculoskeletal Science, University of Oxford, Oxford OX3 7LD, UK.; ^2^ Department of Sports Medicine, Huashan Hospital, Fudan University, Shanghai 20040, China.; ^3^ Department of Engineering Science, University of Oxford, Oxford OX1 3PJ, UK.; ^4^ Devanthro GmbH, 85748 Garching, Germany.; ^5^ Mathematical Institute, University of Oxford, Oxford OX2 6GG, UK.

## Abstract

Mechanical stimulation is essential in tissue engineering and regenerative medicine for proper tissue maturation. However, conventional uniaxial platforms fail to reproduce the multiaxial loading experienced in vivo. In this study, we present a humanoid robotic bioreactor capable of delivering human-like shoulder motions to engineered tendon constructs, enabling controlled multiaxial stimulation with real-time strain monitoring. Human mesenchymal stem cells were cultured on decellularized tendon scaffolds and subjected to adduction–abduction loading at peak strains of approximately 3.5% and 9.5% under external forces of 25 and 50 N, respectively. Strain levels were directly quantified in situ using a flexible sensor integrated within the bioreactor. The transparent bioreactor membrane allowed noninvasive observation while simultaneously applying mechanical stimulation over 14 d, with continuous assessment of cellular morphology without fixation. Compared with static and traditional uniaxial controls, the robot motions enhance cell alignment and activation of mechanotransduction pathways while inducing notable gene and protein expression changes, particularly within the PI3K–Akt signaling pathway. Although dynamic loading resulted in a moderate reduction in cell viability, the transcriptional profile was consistent with mechanically driven phenotypic adaptation toward tenogenic-related programs rather than dominant signatures of acute cytotoxic damage. These findings demonstrate that replicating human-like multiaxial mechanics in vitro fundamentally alters cellular mechanosensing and may provide a mechanobiological foundation for the future development of more physiologically relevant tendon grafts.

## Introduction

Humanoid robots are experiencing rapid advancements, as they offer the prospect of substantial benefits in our daily life such as the automation of repetitive or delicate human tasks. Their potential as tools spans across assistance, caregiving, education, entertainment, research, and exploration [1,2]. Notably, humanoid robot integration in bioreactor systems for engineering biological tissues represents a pioneering application [3,4]. Bioreactors, which maintain and stimulate living cells and tissues by applying mechanical stimulation, are crucial for developing physiologically relevant tissue grafts. In particular, there is increasing evidence that physiologically relevant mechanical cues provided in bioreactors could play a crucial role in addressing musculoskeletal (MSK) repair needs in our aging population [5–7].

Rotator cuff tendon tears are a major cause of shoulder pain in adults, impacting about 20% of the general population [8]. Despite advances in surgical techniques, the failure rate of tendon repairs remains high, with about 40% of patients not achieving satisfactory healing 12 months post-surgery, leading to persistent pain and disability [9]. Tissue engineering offers a promising avenue for repair strategies, yet challenges in replicating the biomechanical environment of tissues have hindered the development of predictive and physiologically relevant engineered tendon grafts [10,11].

Engineered tendons, similarly to native ones, require proper mechanical stimulation in vitro to support cell proliferation, differentiation, and extracellular matrix (ECM) organization, thereby promoting tissue repair and regeneration [6,12,13]. Traditional mechanical stimulation methods, typically involving uniaxial actuation, fail to accurately mimic the complex biomechanics of the human body [14–16]. In tendon tissue engineering, increasing evidence suggests that more complex mechanical environments may influence collagen organization and tenogenic differentiation through distinct mechanotransduction processes [17,18]. However, the biological effects of multiaxial loading remain largely unexplored, as no current platforms can replicate physiologically the relevant motions to determine how different loading modes regulate early mechanotransductive and phenotypic cellular responses. Although tensile strain is recognized as the primary loading mode in the supraspinatus tendon, in vivo studies have reported heterogeneous intratendinous strain patterns and potential shearing between tendon layers during arm elevation [19]. These secondary mechanical components, such as transverse shear or sliding, may contribute to local mechanobiological responses and are not fully captured by conventional uniaxial loading systems. This highlights the need for advanced bioreactor platforms capable of delivering physiologically relevant multiaxial stimulations.

MSK humanoid robots aim to imitate the human MSK system and biomechanics by replicating its anatomical features and utilizing muscle-like units, such as string or pneumatic actuators [20]. These robots can apply multiaxial forces and motion patterns to tissue constructs when integrated with soft bioreactor chambers. Our initial work highlighted the potential of this approach for supporting biological research and medical advancements. However, that elementary platform exhibited limitations in accurately detecting the amount of strain transmitted to the bioreactor during stimulations, despite its critical importance for tissue constructs [21], and measuring strain during complex dynamic motions remains challenging. More importantly, it is not clear that multiaxial stimulation confers distinct mechanobiological effects at the cellular level compared to uniaxial stimulation on traditional platforms. The use of MSK humanoid robots in bioreactor systems therefore requires further validation.

Herein, we investigate the response of human cells cultured on decellularized tendons to the stimulation by a new MSK humanoid robot and uniaxial platform under matched peak tensile strains. To enable this, we develop a flexible and biocompatible strain sensor with in situ strain monitoring capability to address the challenge of strain measurement under multiaxial mechanical conditions. Results show that robot abduction–adduction motions with maximum forces of 25 and 50 N lead to strains of approximately 3.5% and 9.5%, respectively. Notably, the abduction–adduction motions via the robotic shoulder result in significant enhancements in cell orientation, as well as notable changes in gene and protein expression, particularly within the phosphatidylinositol 3-kinase/protein kinase B (PI3K/Akt) signaling pathway, when compared to state-of-the-art static and uniaxial dynamic stimulation controls. These findings emphasize the importance of developing bioreactor platforms that more closely replicate human biomechanics and provide a framework for investigating early cellular mechanosensing under physiologically relevant multiaxial loading conditions.

## Methods

### Construction of robotic shoulder

The MSK robotic shoulder employed in this study was produced by Devanthro GmbH (Garching, Germany), and its design was adjusted to host the soft bioreactor chamber. Hardware components included 8 robotic muscles using polyethylene strings (10lb-300lb PE braided line, Hercules, China) mounted on aluminum rods within 3-dimensional (3D)-printed parts. Each muscle unit consisted of a 25-W brushless EC-max 22 mm in diameter with an encoder and a 53:1 planetary reduction (Maxon Group, Switzerland), a spring, a series of pulleys, an encoder for motor position and spring deflection sensing, and a motor drive board for motor control and encoder reading. The supraspinatus muscle string linked to the soft bioreactor chamber was connected to an s-type load cell sensor (CALT DYLY-108) with an amplifier (PhidgetBridge 4-Input), allowing real-time detection of applied force during stimulation. The motor driver board transmitted the data through a custom RS485-basedbus protocol to a controller board and received pulse width modulation (PWM), velocity, or position setpoints for motor control. The control loop operated at 500 Hz. The controller board, based on a DE10-Nano-SoC development board, integrated a Cyclone V field-programmable gate array (FPGA) with an ARM Cortex-A9 processor. A proportional-integral-derivative (PID) controller on the FPGA regulated motor position, spring displacement (associated with force), or motor velocity. The FPGA system utilized Ubuntu 16.04 with ROS Kinetic as the middleware. High-level kinematic control was implemented on an NVIDIA Jetson Nano board, which utilized Ubuntu 18.04 and ROS Melodic as middleware. A web-based graphical user interface (GUI) facilitated the recording of muscle trajectories, their subsequent replay, as well as data collection. In the shoulder model, the muscle units were utilized to actuate an arm composed of an aluminum rod ending with a spherical plastic ball as the humerus head. The muscle strings were routed from the trunk toward the arm via 3D-printed guides and were attached to a 3D-printed ring fixed onto the aluminum rod. The arm’s ball was fitted into a 3D-printed shoulder socket connected to the trunk’s aluminum frame.

### Strain sensor fabrication

The multi-walled carbon nanotube (MWCNT)-covered Ecoflex fiber (MEF) sensor was fabricated by assembling MWCNTs on a prepared Ecoflex fiber surface and then encapsulating the modified fiber with electrospinning and electrospraying. Using a syringe pump (PHD ULTRA), freshly prepared Ecoflex (0050, from Smooth-On) solutions A and B with the same ratio in a 6-ml syringe were dispensed into plastic tubing (from TFE Teflon) with an inner diameter of 0.8 mm, with the rate of 0.1 ml/min. Subsequently, the Ecoflex in the tubing was placed in an oven with a temperature of 80 °C for 2 h to allow the Ecoflex to cure. The Ecoflex fiber was then obtained by peeling off the plastic tubing. Before modifying the fiber, 50 mg of MWCNTs (from Sigma-Aldrich, >90% carbon basis; *D* × *L* 110 to 170 nm × 5 to 9 μm) in 15 ml of cyclohexane (from Thermo Scientific, ACS, 90+%) was dispersed through ultrasonic treatment using an ultrasonic processor (model W-225) for 10 min. We employed an output control of 5 and a duty cycle of 50% during the process. Cyclohexane was added during the process to maintain consistent volume and concentration. The Ecoflex fiber, cut into a length of 7 cm, was then immersed into the prepared dispersion for ultrasonic treatment with the aforementioned ultrasonic parameters for 1, 3, and 5 min, respectively, before being removed. By contrast, the Ecoflex fibers without ultrasonic treatment were only immersed into the prepared dispersion before being taken out. The MWCNT-modified Ecoflex fiber was placed in a fume hood overnight to finally accomplish the assembly.

MEF, subjected to ultrasonic treatment for 3 min, was cut into approximately 4.4-cm lengths. Both ends of the fiber were sequentially covered with silver conductive paint (SCP03B, from RS) and aluminum foil. This process was carried out step by step in preparation for the subsequent encapsulation of electrospinning and electrospraying. One side of the aluminum foil was connected to a steel wire (100 mm in diameter), while the other side was connected to a piece of polyester thread. The fiber was maintained parallel to the ground without stretching. The steel wire connected to the ground rotated 3 reversible turns along the fiber axis at 40° per second, manipulated by a homemade device as described previously [22]. The nozzle parallel to the modified fiber for electrospinning and electrospraying was carried by a motion control device (ZABER, T-LSR300D) to achieve the encapsulation. The electrospinning solution was prepared by dissolving thermoplastic polyurethane (TPU) (supplied by Permail Gloucester Limited) into 1,1,1,3,3,3-hexafluoroisopropanol (HFIP) (from Halocarbo, >99 wt %) with a concentration of 80 mg/ml on a roller mixer (Stuart, SRT9D) for 24 h with the speed of 60 rpm, while the electrospinning solvent was pure hexafluoroisopropanol (HFIP) . The distance between the nozzle and the collector (i.e., MWCNT-modified fiber) was maintained at 15 cm. During the electrospinning and electrospraying processes, the applied voltages were set at 8 and 7.5 kV, respectively. The extruded velocity of the TPU solution and HFIP was 0.05 and 0.15 ml/min, respectively. The nozzle moved for 20 cycles during electrospinning and 30 cycles during electrospraying, with a speed of 5 mm/s for both processes. The MWCNT-modified Ecoflex fiber underwent electrospinning encapsulation initially, followed by electrospraying to melt the spinning fibers at the outermost layer. Subsequently, the sensor was finally fabricated by allowing it to rest in room conditions overnight.

### Electromechanical characterization of the sensor

The electrical performance of the sensor was measured using a multimeter (Keithley 2400). Programming software, developed by Beijing Hanlei Technology Co. Ltd., was employed when continuous data recording was necessary. Mechanical performance and applied mechanics to all samples were executed using a controllable mechanical instrument (Zwick, Roell Group, Ulm, Germany). The test length for both the MWCNT-modified Ecoflex fibers and sensors was maintained at 20 mm for electromechanical property tests. The surface morphology of the samples was examined with a scanning electron microscope (SEM) (Zeiss Sigma300). The element content and distribution were analyzed using an instrument equipped with energy-dispersive x-ray spectroscopy (EDS). SEM images of the samples under stretching conditions were acquired by stretching and affixing them onto specimen stubs using conductive tapes before measurement. To observe the samples’ cross-sections, whether MWCNT-modified Ecoflex fibers or sensors, they were cut perpendicular to the fiber’s axis using a disposable scalpel (from Swann-Morton). Images of the MWCNT-modified Ecoflex fibers, taken immediately from the dispersion after 1, 3, 5, and 10 min, were captured using a microscope (Olympus BX40). To verify the electrical insulating capacity of the encapsulation layer achieved through electrospinning and electrospray, the MWCNT-modified Ecoflex fiber, a sensor without stretching, and a sensor with a strain of 100% were immersed in phosphate-buffered saline (PBS; 1×) contained in a plastic container, with both ends connected to 2 electrodes. The length of all the samples was approximately 5 cm. A power supply (MASON, EP-603) was connected to the electrodes, and the voltage was applied from 0 to 30 V. During the testing period, a multimeter (Keithley 2000) was utilized to measure the current through another pair of electrodes.

### MEF simulation

For the simulation of electrical changes upon stretching, a pin joint was applied to the fixed end, while a roller boundary condition was applied to the stretching end. A 100% stretch was imposed using Dirichlet boundary conditions at one end. An implicit static solver was used. Spatial convergence was checked, and an element size of 0.001 mm or smaller was used for the simulation. An Ogden hyperelastic model was used for both the outer shell and inner core [23]. The parameters for the Ogden hyperelastic model are shown in Table S2. In this table, *μ*_*i*_​ represents stiffness contributions from different deformation modes, while *α*_*i*_ controls the nonlinearity of the stress–strain response. Together, they allow the model to capture complex, large-deformation behaviors in materials. The initial outer radius of CNT-silicone shell *R*_2, initial_ is 4.001 mm, the initial thickness of MWCNT-silicone shell *h*_initial_ is 0.001 mm, the initial inner radius of MWCNT-silicone shell *R*_1, initial​_ is 4.0 mm, the initial length *L*_0_ is 36 mm, the final length *L* is 72 mm (100% strain), and the initial resistance *R*_0_ = 58,000 ohms. The resistance (*R*) of MEF is calculated by Eq. (1), and the deformed cross-sectional areas (*A*) are calculated by Eq. (2).

$$A=\pi \left(R_{2}^{2}-R_{1}^{2}\right)$$ (1)

where *R*_1_ is the deformed inner radius and *R*_2_ is the deformed outer radius.

### Preparation of scaffolds

Porcine limbs were obtained from a local abattoir. Fresh flexor tendons were harvested by removing all surrounding tissues. The samples were immediately rinsed in PBS to remove extraneous blood and debris. For decellularization, each tendon sample was immersed in a PBS solution containing 1% sodium dodecyl sulfate (SDS) and 1% Triton X-100 within 50-ml sterile tubes. The process lasted for 48 h at room temperature in an orbital shaker at 20 rpm, refreshing the solution every 24 h. Post-decellularization, the samples were washed 3 times for 30 min in distilled water at 4 °C. Afterward, rinses in PBS for 24 h expunged residual SDS, succeeded by three 30-min washes in distilled water at 4 °C. Final scaffold sterilization utilized 70% ethanol for 1 h, ensuring asepsis for subsequent applications. After sterilization, the solution was removed, and the decellularized tendons were washed 3 times with PBS for 30 min each to fully cleanse the material. After decellularization, each tendon sample was then precisely cut to dimensions of 28 mm in length, 5 mm in width, and 0.3 mm in thickness. Fiber bundles of polycaprolactone (PCL) and polydioxanone (PDO) with aligned configurations were prepared through the approaches reported in our previous work [22,24]. Poly-Tapes were donated by Xiros Ltd.

### Fabrication of the 3D-printed chamber components

Designs for the chamber components were created using Inventor Professional 2023 (Autodesk, San Francisco, CA, USA). These components were then 3D-printed using BioMed Amber resin (Formlabs, Massachusetts, USA) in a Form 3B+ 3D-printer (Formlabs, Massachusetts, USA). The printed components comprised a main insert for anchoring the scaffold, an intermediate ring to hold the membrane, and an end plate for securing the membrane onto the main insert. Rubber O-rings (Polymax, Bordon, UK) were fitted into the grooves of the main insert and the inside of the endplate to ensure the chamber’s leak-tight integrity.

### Fabrication of the flexible tubular membrane

The flexible tubular membranes were crafted from a transparent, polyether-based TPU film (TFL-2EA, 50 μm thickness, donated by Permali Gloucester Limited, Gloucester, UK). This film was folded in half, and its long edge was seamlessly welded using an electric heat sealer (Cole-Palmer, Illinois, USA) to achieve a precise internal width of 16 mm. After welding, the tubes were trimmed to a uniform length of 7 cm. For assembly, each end of the tubular membrane was aligned and affixed onto an intermediate ring. This was done by threading the membrane through the ring’s hole and wrapping its inner surface around the outer circumference of the intermediate ring. After attaching the membrane to the rings, the assembly was then inserted into an endplate by carefully guiding the rings through the specifically designated holes in the endplate. The total length of the tubular membrane, once stretched between the 2 endplates, was carefully adjusted to approximately 30 mm.

### Fabrication of the soft bioreactor with strain sensing function

The assembly of the chamber commenced with the insertion of the decellularized tendon into the hole of the first main insert. To ensure structural integrity and prevent the capillary effect in the materials, Epoxy resin (Epotek 301, Epoxy Technology Inc., Billerica, USA) with a relatively high viscosity was injected into the hole. This resin was allowed to cure for 24 h to ensure a solid set. Subsequently, the free ends of the scaffold were threaded through the tubular membrane and its supporting components. They were then positioned into the hole of the second main insert, where another dose of epoxy resin was applied and left to set for an additional 24 h. Following this, polytetrafluoroethylene (PTFE) tubing [1/16 outer diameter (OD), inner diameter (ID) 0.8 mm, Sigma-Aldrich, Dorset, UK] was incorporated at both ends of thechamber through dedicated channels. Epoxy resin was also applied around these junctions to guarantee a leak-proof seal. Additionally, small loops of PE braided cord (Hercules, China) were affixed to both ends of the chamber. One loop was connected to the humeral shaft of the humanoid shoulder arm, and the other to the load cell linked to the supraspinatus muscle string.

### Cell preparation and cell culture

Green fluorescent protein (GFP)-expressing mesenchymal stem cells (MSCs), prepared according to the method of Mihara et al. [25], and GFP-expressing human dermal fibroblasts (H-6068L, Cellbiologics, Chicago, USA) were cultured in Dulbecco’s modified Eagle’s medium (DMEM) supplemented with 10% fetal bovine serum (Biosera, Heathfield, UK) and 1% penicillin–streptomycin solution within appropriate culture flasks. Incubation was carried out at the standard conditions of 37 °C with a 5% CO_2_ atmosphere, and the growth medium was refreshed every 2 d. Cellular morphology and confluence were routinely examined under a microscope. The cells were subcultured when they achieved 70% to 90% confluence.

Before cell seeding, all chambers underwent sterilization by injecting 70% ethanol into each chamber for 2 h, followed by a triple wash with PBS. For cell culture within the chambers, confluent flasks were trypsinized, and cells were gently scraped off, centrifuged, and then resuspended in the appropriate culture medium. Approximately 0.5 × 10^6^ cells in 200 μl of growth medium were introduced into each chamber using 1-ml syringes equipped with blunted needles (18-gauge, Temuro Ltd., Surrey, UK). An additional 100 μl of growth medium was carefully injected to ensure complete cell introduction into the chambers, minimizing any residual cells in the delivery system. After a 60-min incubation period, 1 ml of fresh medium was introduced to the chambers. Subsequently, the culture medium was refreshed every 2 d.

### Dynamic stimulation on the humanoid robotic shoulder and uniaxial platform

After seeding each bioreactor chamber, they were cultured in static conditions for 2 d before introducing mechanical stimulation from day 3 to day 14, spanning a total of 12 d. Throughout the culture period, daily mechanical stimulations were conducted on the chambers using a humanoid shoulder arm or a uniaxial platform. For the dynamic stimulation on the humanoid robotic shoulder, before mounting the chambers onto the humanoid shoulder, a specific sequence of 2 adduction and abduction movements, each spanning approximately 60° from a reference position parallel to the robot’s trunk, was manually applied to the arm. The tension in the springs on the humanoid robotic shoulder and the uniaxial platform (the actuators attached to the chambers) was carefully adjusted to reach a predefined maximum force, which could be either 25 or 50 N. Once the loading parameters were configured, the chambers were transferred to both platforms, situated outside the incubator. To ensure an adequate supply of nutrients during stimulation, approximately 1 ml of medium was retained within each chamber. The adduction–abduction exercises on the humanoid robotic shoulder or the stretching exercises on the uniaxial platform were repeated for 30 min at a frequency of approximately 0.08 Hz for both platforms. Deformations of the scaffold within the chambers, where cells adhered, were continuously monitored using strain sensors. The stimulation on the uniaxial platform was performed by an X-LSM025A-E03 motorized linear stage (Zaber, Vancouver, Canada), and the stimulation conditions, including time and strains, were kept the same as those on the humanoid robotic shoulder.

### Cell viability assessment

PrestoBlue assay (Invitrogen, Paisley, UK) was employed to evaluate cell viability within the chambers. The culture medium was removed from the chambers. The chambers were washed with PBS and then replaced with 1,000 μl of a 10% PrestoBlue solution (v/v in DMEM). Following a 1-h incubation at 37 °C, PrestoBlue medium samples were decanted from each chamber into sterile Eppendorf tubes. Subsequently, 100 μl of these samples was transferred to white 96-well plates (Corning, UK) for analysis, with each sample analyzed in triplicate. A blank control of 10% PrestoBlue solution was also included. Fluorescence measurements were carried out using the FluoStar Optima microplate reader (BMG Labtech, Ortenberg, Germany, λex = 544 nm, λem = 590 nm). For cell and scaffold type selection, the assay was performed on days 1, 3, 6, 10, and 14. In the case of mechanical stimulation, the assay was conducted on days 2, 6, 10, and 14. PrestoBlue assays were performed on *n* = 3 independent chambers per condition.

### Biocompatibility assessment of the MEF sensor

MSCs were utilized to evaluate the biocompatibility of the MEF. MEF sensors were cut into 0.5-cm pieces and placed at the bottom of separate wells in a 24-well plate. These sensors were sterilized by immersing them in 70% ethanol for 2 h, followed by 3 washes with PBS. MSCs were then seeded onto the MEF sensors in the 24-well plates at a density of 5.0 × 10^4^ cells, with 1 ml of cell suspension added per well. Seeded wells with no MEF sensors served as the negative control (indicative of live cells), and 100 vol % dimethyl sulfoxide (DMSO) served as the positive control (indicative of dead cells).

Cell viability was assessed using both PrestoBlue cell viability assay and flow cytometry live/dead assay after 24, 48, and 72 h. The procedure for the PrestoBlue cell viability assay was conducted as previously described. For the flow cytometry live/dead assay, cells were first detached from the MEF sensor using trypsin, then washed and resuspended in a staining solution containing propidium iodide (PI) for dead cells and fluorescein diacetate (FDA) for live cells. This mixture was incubated for 20 min at room temperature in the dark. After incubation, cells were washed and resuspended in PBS for analysis. The samples were then analyzed using a flow cytometer (BD Fortessa, BD Biosciences, USA), where the percentage of live cells was determined and quantified in the Q3 quadrant, illustrating the proportion of live versus dead cells within the population.

### Force and strain sensing during stimulation

Force was measured using a force sensor located between the bioreactor chamber and the robotic muscle unit, as well as the uniaxial device. The strain was determined by recording the changes in resistance of the strain sensor during stimulation and by transforming those into strain values from a calibration curve. Calibration was performed for each chamber after embedding the sensor and the decellularized tendon, and we applied a strain of up to 15% to the constructs. During biological experiments, we collected force and resistance (strain) data for 10 cycles within each 30-min session, and the frequency of one adduction–abduction motion is 0.08 Hz. We initiated and terminated the recording of both datasets simultaneously. During data analysis, the force and strain data were aligned through the interpolation/extrapolation function of OriginLab 8.5 using time as a reference variable.

### Confocal microscopy

Cells were observed on days 0, 2, 6, 10, and 14 using a confocal laser scanning microscope, with mechanical dynamic stimulation initiated on day 2. Following the PrestoBlue assay, approximately 400 μl of culture media was retained within each chamber to facilitate optimal imaging conditions. The chamber was carefully placed into a custom chamber holder (Fig. S30), and gentle pressure was applied with fingers to ensure that both the chambers and the scaffolds were evenly pressed downward, securing them in place. Imaging was conducted at a temperature of 21 °C using an inverted Zeiss 880 microscope equipped with an Airyscan detector. For the visualization of GFP-labeled cells, a 488-nm argon laser was employed. The pixel size was set to 1.66 μm, the detection pinhole was 1 airy unit (AU), and the bit depth was 8 bits. For detailed images, a Z-stack with 0.5-μm intervals encompassing 20 layers was collected. Images showing maximum intensity were then processed using ImageJ software. Processing included generating a Z-projection and setting a scale bar.

### Cell morphology and orientation analysis

The changes in cellular morphology and orientation due to stimulation by different mechanical conditions were quantified using ImageJ software (National Institutes of Health, Bethesda, MD, USA). For analysis, each image was divided into 5 quadrants, and 10 cells from each quadrant were selected. Measurements were obtained from 3 distinct images from each chamber.

To analyze the cell morphology, the cell aspect ratio (major axis length/minor axis length) was obtained. The outer profile of each cell was manually outlined and fitted with the closest-fitting ellipse to determine the major and minor axes, representing the cell’s length and width, respectively. The aspect ratio was used as an index to express the degree of cell elongation in response to the underlying microstructures.

To quantify the cell orientation, the angle between the major axis of the cell and the direction of stretch was measured. Cellular orientation was defined as the angle between the major axis fitted within the ellipse profile and the vertical orientation of the microstructures. The orientation angles were divided into 3 groups: 0° to 30°, 31° to 60°, and 61° to 90°. This categorization allows for a detailed analysis to determine if there are significant differences among these groups.

### Bulk RNA sequencing

Constructs were harvested in 500 μl of Trizol (Sigma-Aldrich, Dorset, UK). Samples were centrifuged at 13,000 rpm for 8 min at 4 °C, and the supernatant was collected. RNA was extracted using Zymo Direct-zol RNA Miniprep Plus Kits (Cambridge Biosciences, UK) according to the manufacturer’s protocol. RNA concentrations in the eluted samples were determined using a Nanodrop Machine (Implen GmbH, München, Germany). The preparation of RNA library and transcriptome sequencing was conducted by Novogene Co. Ltd. (Cambridge, UK). Library preparation was done using a NEBNext Ultra II Directional RNA Library Prep Kit for Illumina with poly-A selection (Illumina, San Diego, CA, USA) following the manufacturer’s instructions. For library preparation, 100 ng of RNA was used. cDNA content of every library was quantified using a BioAnalyzer (Agilent, Santa Clara, CA, USA). Libraries were pooled and run on an Illumina NovaSeq 6000 using the 75 cycles NextSeq High Output kit (Illumina).

The original image data file from the high-throughput Illumina sequencing platform was transformed into raw reads by the Illumina Casava (v1.8). These raw data were stored in FASTQ format files, which contain sequences of reads along with their corresponding base quality. Quality assessment of raw reads was accomplished using FASTQC and ReadQC tools. Alignment of raw reads to the GRCh38 reference genome was achieved using hisat2 (v 2.0.5). Visualization of the mapped reads was conducted using IGV (v2.3.74) to further evaluate the mapping quality. The quantification of mapped reads against GCRh38 reference genome annotation was carried out using FeatureCounts (v1.5.0-p3), and then FPKM of each gene was calculated based on the length of the gene and reads count mapped to this gene. Subsequent analyses were completed using R 4.2.2. Differential expression analysis was performed using EdgeR (v 3.28.1) by using quasi-likelihood (QL) *F* tests. Genes with adjusted *P* value < 0.05 and |log_2_(foldchange)| ≥ 1 were determined as significantly differential expressed. Gene Ontology (GO) and Kyoto Encyclopedia of Genes and Genomes (KEGG) analysis was performed using the enrichGO and enrichKEGG function of the clusterProfiler 3.14.3 package, respectively, where categories with *P* ≤ 0.05 were considered significantly enriched. Heatmaps and volcano plots were visualized by using pheatmap 1.0.12 and EnhancedVolcano 1.4.0, respectively.

### Western blot

All reagents were supplied by Thermo Fisher Scientific, UK. Total protein extraction from the samples was performed using radioimmunoprecipitation assay (RIPA) buffer, and protein concentrations were quantified using the BCA Protein Assay Kit. Proteins were normalized and prepared for gel electrophoresis by mixing with Novex Sample Buffer. Separation was achieved using Novex Protein Gels, followed by transfer onto 0.45-μm polyvinylidene fluoride (PVDF) membranes using the Bio-Rad Vertical Electrophoresis Cell and Trans-Blot Transfer System.

For protein detection, membranes were first blocked with protein-free blocking buffer for 1 h at room temperature. This was followed by overnight incubation at 4 °C with primary antibodies. After washing with 1× tris-buffered saline Tween buffer, membranes were incubated with goat anti-rabbit immunoglobulin G (IgG) for 1 h at room temperature. Protein bands were visualized using SuperSignal Western Blot Enhancer and captured with a Bio-Rad Multi Fluorescence imager. The specific antibodies employed in the Western blot analysis are detailed in Table S3. Protein analysis was performed using *n* = 3 independent constructs per condition.

### Statistical analysis

Quantitative data, including the sample’s conductivity, strain deformation of the strain sensor, PrestoBlue fluorescence intensity, and relative protein expression levels, were presented as mean ± standard deviation. The resistance measurements were conducted on a sample size (*n*) of 5, with each sample having a length of 5 cm. The strain deformation analysis involved a subset of the samples *N* = 3, and a repeated measurement (*n* = 3) was undertaken. For statistical comparison, *t* tests or 2-way analysis of variance (ANOVA) was performed to identify significance between groups. Statistical significance was established at *P* < 0.05. All statistical analyses were performed using GraphPad PRISM version 8 software (GraphPad Software Inc., La Jolla, CA, USA).

## Results

### Mechanical stimulation platforms and experimental concept

**Fig. 1.**
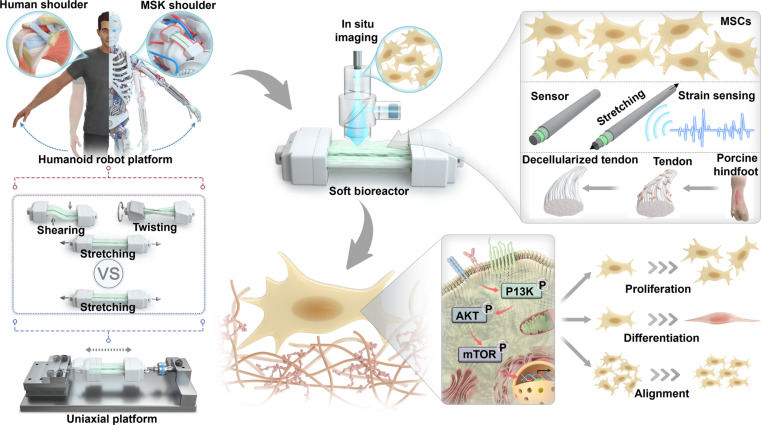
Conceptual illustration of the experimental framework used to investigate cellular responses to human-like multiaxial loading. The MSK humanoid robot delivers shoulder-like motion to cell-seeded decellularized tendon scaffolds housed within a soft bioreactor. The chamber incorporates a fiber-based strain sensor for real-time deformation monitoring and a transparent membrane to allow noninvasive imaging of cellular morphology. Unlike conventional uniaxial platforms that apply only linear stretching, this platform allows direct comparison of cellular behaviors, such as proliferation and alignment and differentiation under multiaxial versus uniaxial stimulation, providing insight into the mechanotransduction pathways activated by physiological motion.

As illustrated in Fig. 1, the humanoid robotic bioreactor provides a controlled platform to compare multiaxial and uniaxial mechanical stimulation of MSCs. Unlike conventional uniaxial systems, which apply only linear tensile strain, the robotic platform can reproduce human-like joint motions to apply complex mechanical stimulation such as stretching, twisting, and shearing. MSCs were cultured on decellularized porcine tendon scaffolds housed within a soft bioreactor chamber that integrates a fiber-like strain sensor assembled through layer-by-layer techniques. The transparency of the chamber membrane facilitates live cell observation via confocal microscopy without interrupting culture. By exposing cells to physiologically relevant mechanical cues, this approach enables investigation of how complex loading environments influence proliferation, alignment, and early tenogenic differentiation compared with conventional uniaxial loading.

### Soft sensor design and fabrication

**Fig. 2.**
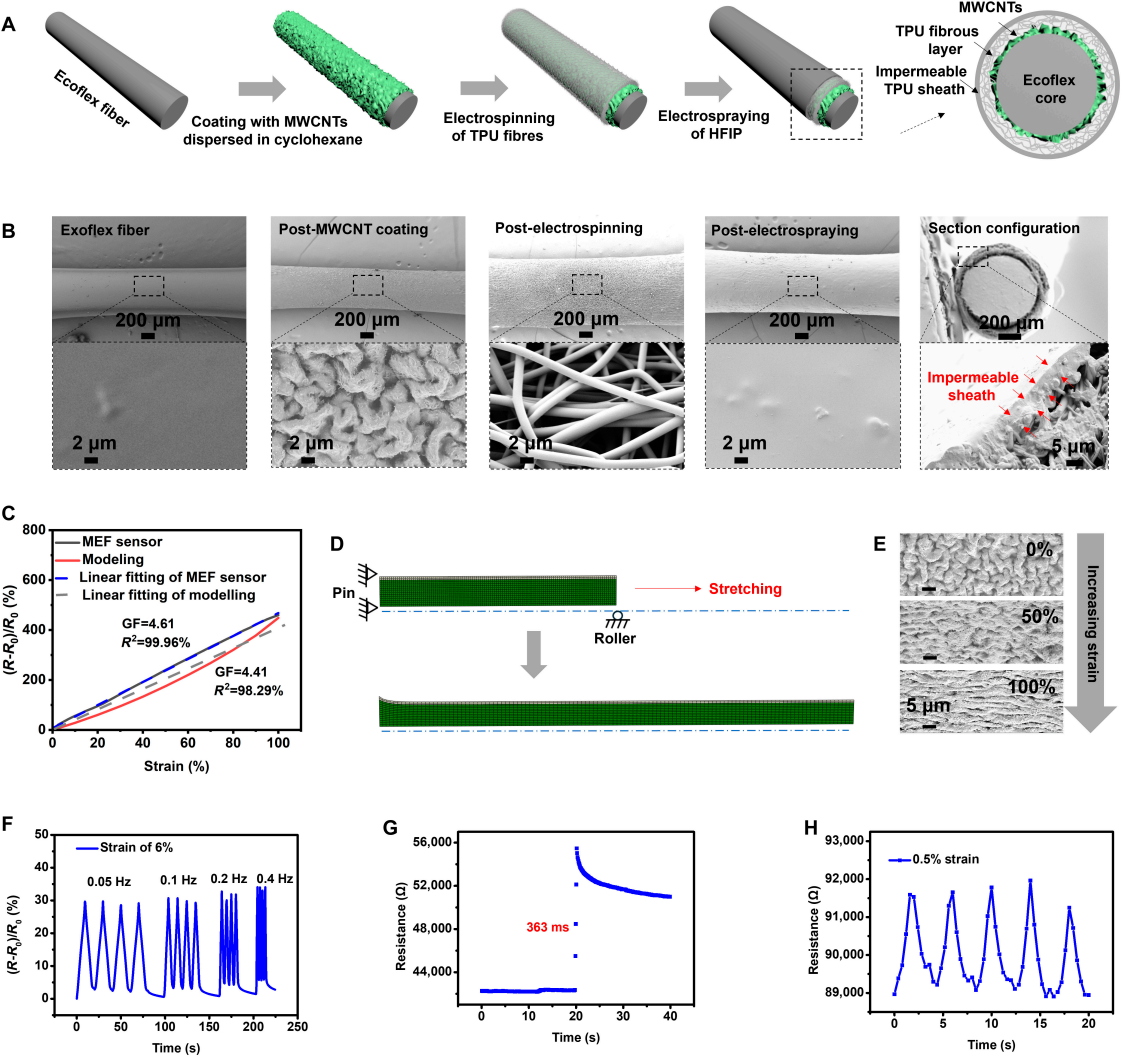
Fabrication and electromechanical performance of the MEF strain sensor. (A) Step-by-step illustration of the sensor fabrication. (B) Sequential morphology of the sample during sensor fabrication. (C) Typical calibration of the sensor and the simulated electrical outputs of the (D) model, showing the sensitivity and linear sensing feature. *R*_0_ and *R* denote the initial resistance and the resistance after stretching of the sensor, respectively. (D) Axisymmetric finite element model of MEF. The white part is the MWCNT-silicone shell, and the green part is the silicone core. The dashed line corresponds to the central axis. Roller supports were applied to the 2 end surfaces of the sensor, and 50% strain displacement was applied to the same surfaces. (E) Surface morphology of MEF upon stretching. (F) Relative resistance changes of the sensor with repeat strain of 6% under various frequencies to show its dynamic stability. (G) Resistance response of the sensor upon applying a quasi-transient step displacement of 1 mm at the speed of 8 mm/s, showing a fast response time of 363 ms. (H) Real-time resistance of the sensor during a cyclic 0.5% strain applied after stretching the sensor to a strain of 20% to show the low detection limit.

As indicated in Fig. 2A, the strain sensor was produced by modifying an Ecoflex silicone elastomer fiber with dispersed MWCNTs in cyclohexane onto the fiber surface (Fig. S1), and subsequently encapsulating it with TPU on the outermost surface through electrospinning and electrospraying (Fig. S2). The resulting sensor displays a core-sheath configuration with the conductive MWCNT layer and the impermeable TPU layer as sensing and isolation elements, respectively.

Achieving a stable coating of Ecoflex fibers with MWCNTs is essential for optimal sensor performance. MEF exhibits enhanced conductivity with increasing treatment time and in the presence of ultrasounds (Fig. S3). Ultrasounds amplify the swelling of the fiber in cyclohexane, consequently resulting in more MWCNTs being embedded on its surface and in higher conductivity (Fig. S4). The treatment also led to numerous wrinkles being formed on the surface (Fig. S5A). MWCNTs contributed to their formation, since pristine Ecoflex fibers are smooth and maintain their morphology after ultrasonic treatment in pure cyclohexane (Fig. S5B). In the presence of MWCNTs, wrinkles appear at the surface of the fibers within 1 min and increase with a longer ultrasonic time (Fig. S6). A proposed mechanism of MWCNT assembly on Ecoflex fiber is shown in Figs. S7 to S9. In this work, we selected an MEF sensor produced with an ultrasonic treatment of 3 min.

As shown in Fig. 2B, a noticeable layer of randomly arranged TPU microfibers envelops MEF after electrospinning. The outermost layer of these microfibers was dissolved upon electrospraying of pure solvent to form an impermeable layer. The final size of the sensor is 44 mm in length and 0.9 mm in diameter. Achieving such an impermeable layer is needed for the function of the sensor and for preventing harmful effects on cells, which may have been caused by passing current during strain sensing. A viability assay indicates that the developed sensor is biocompatible (Fig. S10).

### Soft sensor performance

The sensitivity of the sensor was calculated under the strain of 100%, considering that strains applied in tissue engineering are typically below 10% [26,27]. The influence of the ultrasonication treatment on gauge factor (GF) is shown in Fig. S11. Importantly, the sensor exhibits a linear sensing capability throughout the sensing range, with and without TPU encapsulation. It is found that TPU encapsulation does not affect sensitivity (Fig. S12). The calibration in Fig. 2C indicates that the MEF sensor has a GF of 4.61, and the simulation shows a similar outcome in terms of sensitivity and linear sensing feature. We utilized an axisymmetric finite element model composed of 4 node bilinear quadrilateral elements for the core and shell (Fig. 2D) to simulate the deformation characteristics of MEF under various strain levels. We extracted the deformed dimensions of the MWCNT-silicone shell, focusing on the thickness and inner radius measurements at various strains. Using these measurements, we calculated the cross-sectional area of the MWCNT-silicone shell before and after deformation. The resistance (*R*) of MEF was then determined using Eq. (2). The results show that the electrical signals increase with applied strain with high linearity, consistently with the experimental behavior of the sensor under deformation. This confirms the linear sensing feature. The slight differences between the simulation and experimental results could be attributed to the simplified model being not exactly the same as MEF. Detailed simulation configurations are clarified in Methods.

$$R=R_{0}\frac{L}{L_{0}}\frac{A_{0}}{A}$$ (2)

where *R*_0_ is the initial resistance, *L*_0_ and *L* are the initial and final lengths, and *A*_0_ and *A* are the initial and deformed cross-sectional areas, respectively.

SEM images show that the gradual separation of surface wrinkles transitions to a flat state upon the application of stretching (Fig. 2E). As the strain intensifies, the embedded MWCNTs could progressively part ways, leading to elevated resistances in both contact and noncontact configurations (tunneling effect), consequently raising the total resistance (Fig. S13).

Furthermore, we find that the MEF sensor exhibits a fast response, high detection limit, durability, and good stability under static and dynamic conditions. By applying a strain of 6% at different frequencies (i.e., 0.05, 0.1, 0.2, and 0.4 Hz), the relative resistance changes of the sensor show an immediate response with high consistency (Fig. 2F). Similar outcomes occur when applying different strains (i.e., 5%, 25%, 50%, and 75%) to the sensor at a fixed frequency of 0.1 Hz (Fig. S14A). However, it is worth noting that the electrical signals do not recover to the initial state, and that the declines become more pronounced under higher strains. Similarly, the sensor’s response to a strain of 20% exhibits a more distinct overshoot when the strain rate is higher (Fig. S14B). To understand these findings, we applied a cyclic strain of 40% to the sensor at a speed of 0.67 mm/s. A mismatch in relative resistance changes during the loading and unloading of strain can be observed (Fig. S14C). This mismatch is the electrical hysteresis that originates from the viscoelasticity of the Ecoflex, which is consistent with previous work [28]. We evaluate the response speed of the sensor by applying a quasi-transient step displacement of 1 mm at 8 mm/s. The response time reaches 363 ms from the high-resolution time–resistance curve (Fig. 2G), which is high enough to detect many motions. Fig. 2H displays the real-time resistance of the sensor during a cyclic 0.5% strain applied after stretching the sensor to a strain of 20%. The sensor has a low detection limit down to a strain of 0.5%. One particularly important property is that the sensor acquires high durability during cyclic loading and unloading for 1,600 cycles (Fig. S14D), except for incipient overshot resulting from the viscoelasticity of Ecoflex as well. To confirm TPU encapsulation preventing current leaks, we utilized a multimeter (Fig. S15A), a power supply (Fig. S15B), and a container with a PBS solution (Fig. S15C). The power supply and multimeter were employed to apply current to the testing sensors and evaluate the current in the PBS, respectively. The results show detectable current in the PBS for the nonencapsulated MEF, while no detectable currents for the TPU encapsulated MEF sensors, even under a stretching strain of 100% (Fig. S15D). This indicates that the range of strain explored does not destroy the surface impermeable structure of the MEF sensor (Fig. S16).

### Sensor integration and scaffold selection

**Fig. 3.**
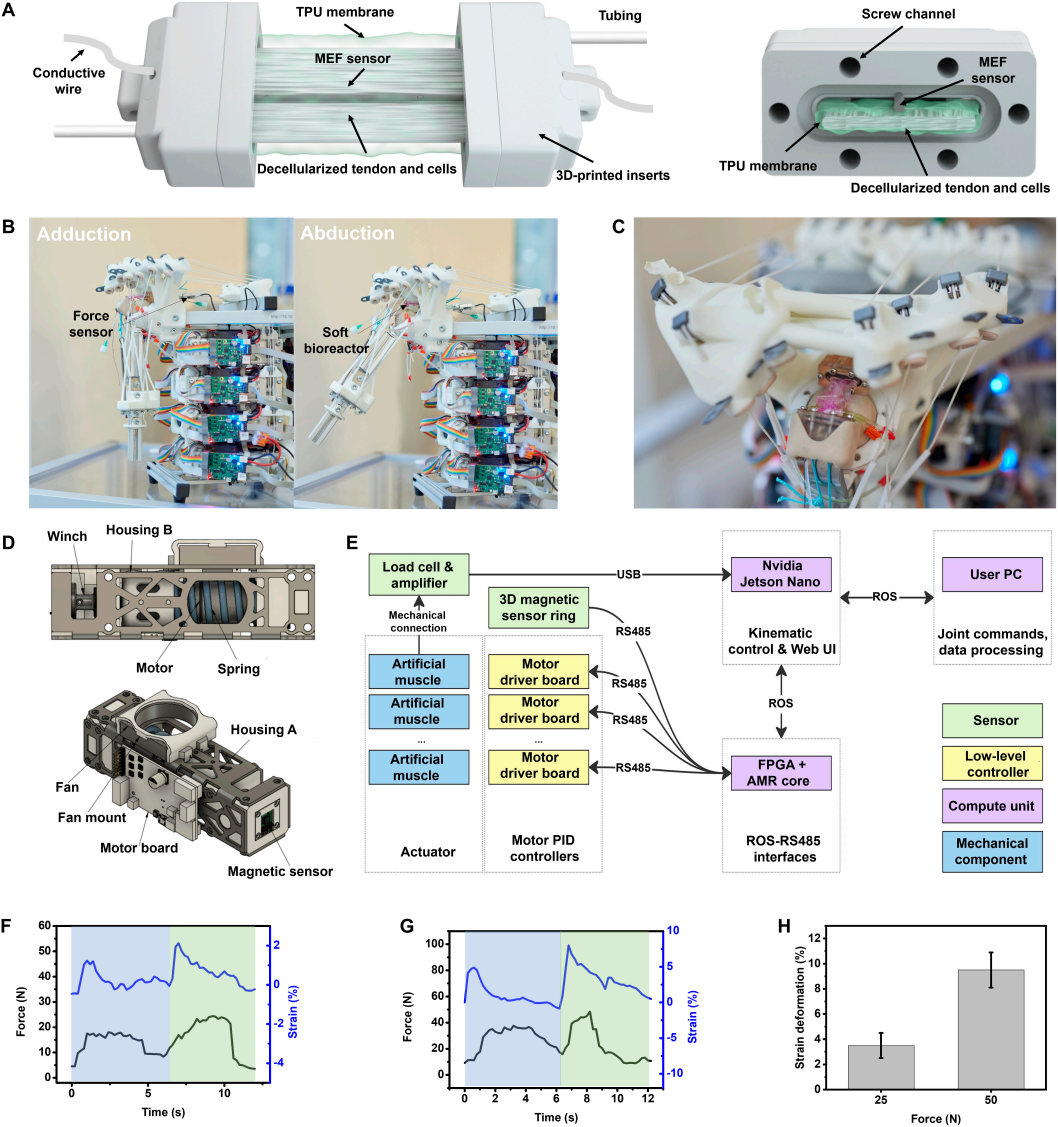
Mechanical stimulations to cells in the soft bioreactor by humanoid robot shoulder. (A) Illustration of (left) soft bioreactor and (right) its section view. (B) Images of the humanoid robot shoulder in adduction and abduction achieved with 8 independent control units and a force sensor connected to the bioreactor to detect mechanical force. (C) Image of soft bioreactor on the robot joint. (D) Construction of the control unit in different views. (E) Illustration of the control framework and movement mechanism of the robot. (F) Typical force and strain curves during one cyclic abduction–adduction motion with the load force of 25 N. (G) Typical force and strain curves during one cyclic abduction–adduction motion with the load force of 50 N. (H) Strain deformation summary during mechanical stimulation with the load force of 25 and 50 N. Data are presented as mean ± SD (*n* = 3).

The MEF sensor is integrated into the soft bioreactor chambers as illustrated in Fig. 3A. Conductive wires are used to connect the MEF sensor at both ends to collect electrical signals for strain sensing during mechanical stimulation, where the sensor is positioned along the centerline of the scaffold’s top surface. The sensor and scaffold were secured in 3D-printed inserts using epoxy resin. Then, they were contained in a thin tubular TPU membrane to create a sterile cell and culture medium environment. Tubing was also fitted in the inserts to enable cell seeding and culture medium replacement every 2 d.

In selecting the optimal scaffold material, a comparative analysis of cell viability and morphology was conducted over 14 d on 4 distinct scaffolds: electrospun PCL, electrospun PDO, Poly-Tape (woven polyethylene terephthalate), and porcine decellularized tendon (Fig. S17). Notably, porcine decellularized tendons emerged as the superior choice, exhibiting a marked increase in cell viability, particularly after day 6, underscoring their exceptional ability to support cellular growth and proliferation (Fig. S18). This observation was further validated through morphological assessments under confocal microscopy, allowing continuous observations within the same chamber. The study employed both human MSCs and fibroblasts, with MSCs showing a notably faster growth rate. Importantly, despite using human-derived cells, the porcine-origin scaffolds demonstrated exceptional compatibility. It is well established that porcine tissues, particularly in the context of ECM, share significant similarities with human tissues [29]. Furthermore, human tendon stem/progenitor cells have been shown to survive and thrive when seeded into porcine decellularized tendon [30], underscoring its compatibility and potential for applications in tendon repair and tissue engineering.

### Robotic shoulder platform

The robotic shoulder used as a mechanical platform in this work is an MSK robotic ball-and-socket joint with 3 degrees of freedom (DOFs). For the present experiments, only the abduction and adduction motion was activated to provide a controlled loading mode to the tendon construct (Fig. 3B). A socket is available on the humerus head for the chamber’s attachment to bone, while the other side of the chamber is attached to the muscle string, via a force sensor (Fig. 3C). In terms of robotic design, the joint is powered by 8 independent artificial muscles using series-elastic actuators, designed to mimic natural antagonistic–protagonistic muscle movements. Each actuator (Fig. 3D) comprises a brushless direct current (DC motor, integrated with a torsional spring, attached to a Dyneema string). Muscle control is managed via a custom motor driver board, featuring a PID controller in PWM and velocity modes, alongside reading encoder feedback for motor position and torsional spring angle measurements. The actuators are powered by a 24-V supply. These boards link to a central processing unit through a proprietary RS485 bus protocol, which coordinates low-level muscle activities. This central unit utilizes a Terasic DE10-Nano Development Kit equipped with a Cyclone V System on Chip Field-Programmable Gate Array and an ARM Cortex-A9 processor (Fig. 3E). In addition to executing PID controllers and processing proprioceptive feedback of the muscles, the FPGA development kit gathers data from an array of TLE493d 3D magnetic sensors arranged on a circular printed circuit board (PCB) around the ball joint via I^2^C interface, facilitating shoulder joint orientation estimations. It also functions as a robot operating system (ROS) node, bridging actuator and joint-level controls, and broadcasting relevant information through ROS topics and services. These data are then consumed by CARDSflow, an open-source framework for the development, simulation, and control of tendon-driven robots. Running on an Nvidia Jetson Nano board, CARDSflow engages with the FPGA through a ROS-based interface over the RJ45 local area network (LAN). It dynamically computes muscle lengths based on the robot’s kinematics and desired joint trajectories, which can be inputted through a web GUI or programmatically via a ROS topic. The GUI also enables the recording and playback of joint and muscle movements by manually guiding the arm in a compliant mode as well as replaying them in a user-defined sequence (e.g., pauses and number of repetitions). The web GUI also serves as a front-end to record and access experimental data, which include time-stamped load cell values and joint poses. The FPGA and Jetson Nano are powered by a 5-V supply.

### In situ sensor measurements during mechanical stimulation

Bioreactor chambers were subjected to 2 different force regimes, 25 and 50 N, based on peak forces. Mechanical stimulation started on the third day after cell seeding, following our previous work demonstrating that low-frequency adduction–abduction loading supports tenogenic differentiation without inducing cellular fatigue [3]. Accordingly, single chambers underwent adduction–abduction cycles at 0.08 Hz for 30 min per day until day 14. For each stimulation period, force and electrical data were recorded over 10 cycles. Fig. 3F and G shows typical force–strain data with the load force of 25 and 50 N, respectively. The strain deformation was calculated according to the calibration of the MEF sensor within each bioreactor. It should be noted that the force required by the sensor to achieve relevant strains was found to be 2 orders of magnitude lower than the decellularized tendon and that, therefore, its contribution could be neglected (Fig. S19). This observation reinforces the sensor’s capability to accurately reflect the deformation of the cell–material construct under mechanical stimulation. A consistent relationship was observed between the applied force and strain deformation, with increased force substantially increasing the strain deformation. It is noteworthy that there were 2 distinct peaks within each adduction–abduction motion, regardless of the force regime applied (25 or 50 N). The force and the strain were observed to rise synchronously, reaching the first peak during slight arm abduction (Fig. S20A) and a second peak upon arm return from maximum abduction (Fig. S20B). The observed dual peaks are primarily attributed to the hysteresis inherent in the robotic system. The system operates on a principle where each control unit targets a specific string length, yielding sensor readings that incorporate both the real-time length of the string and the degree of spring compression. This methodology introduces several nonbiological constraints, including hysteresis arising from friction in string routing, string elasticity, and bandwidth limitations stemming from the motors [31]. Our analysis reveals that applying loads of 25 and 50 N induced peak strains of 3.5% and 9.5%, respectively (Fig. 3H and Figs. S21 and S22).

### Cellular responses to mechanical stimulation: Noninvasive microscopy and viability

**Fig. 4.**
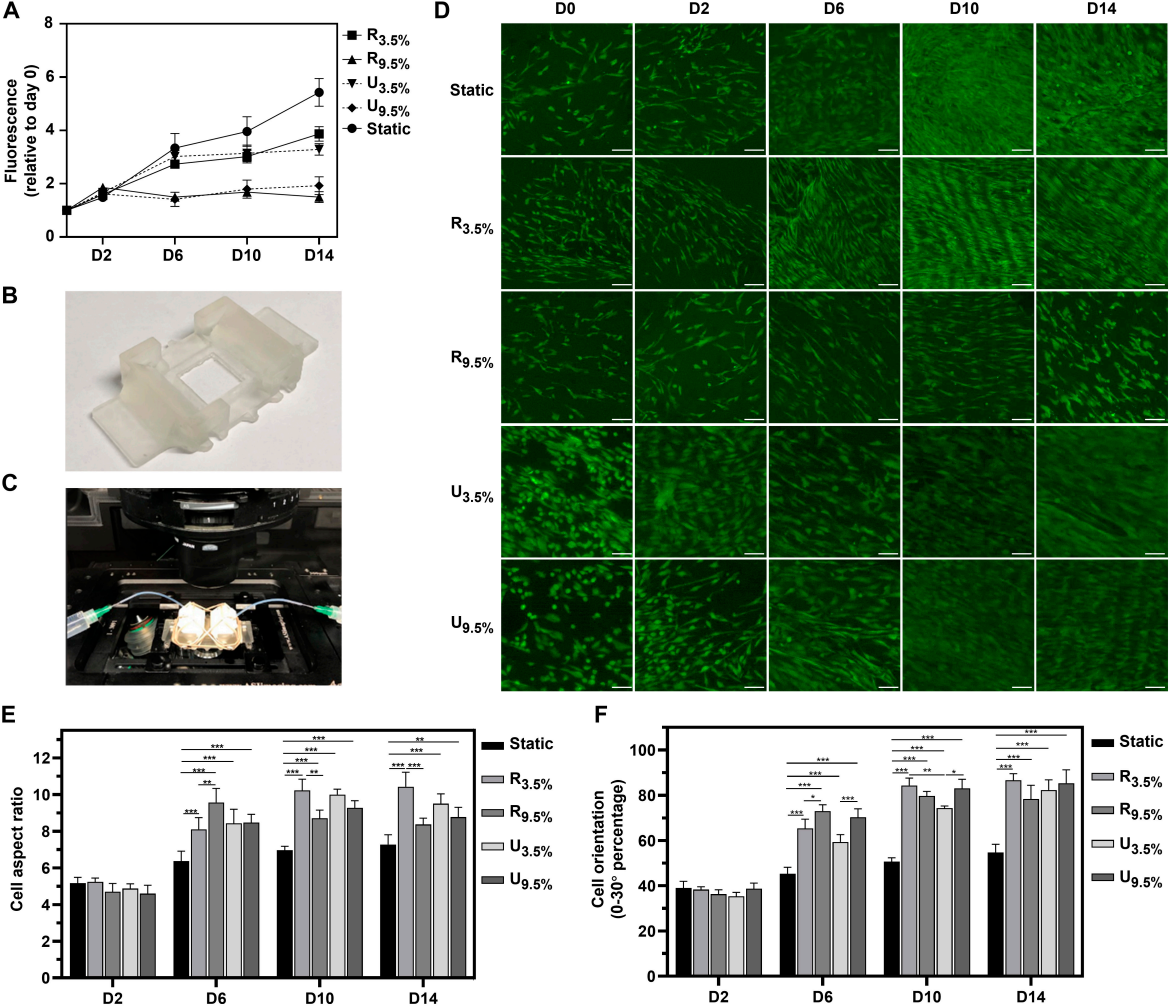
Cellular responses to mechanical stimuli: noninvasive microscopy and viability. (A) PrestoBlue viability assays of MSCs under static, uniaxial, and robot stimulation at different strains. (B) Bioreactor chamber mount for noninvasive confocal microscopy imaging. (C) Microscope setup with a TPU membrane-mounted bioreactor chamber. (D) Time-lapse confocal images of MSCs with GFP green fluorescence captured on days 0, 2, 6, 10, and 14 under static conditions and uniaxial and robot stimulation at different strains. Scale bar, 50 μm. (E) Analysis of MSC cell aspect ratio (major axis/minor axis), captured on days 2, 6, 10, and 14. (F) Analysis of MSC cell orientation captured on days 2, 6, 10, and 14. The percentage of cells with orientation angles from 0° to 30° was specifically quantified to assess alignment in response to mechanical stress. Data are presented as mean ± SD (*n* = 3). Statistical significance was determined using 2-way ANOVA (*P < 0.05; **P < 0.01; ***P < 0.001).

PrestoBlue viability assays indicate that, within the robotic platform, cells subjected to strains of 9.5% (R_9.5%_) and 3.5% (R_3.5%_) exhibit statistically reduced viability compared to those in static culture, with R_3.5%_ stimulation showing intermediate viability levels (Fig. 4A). This is in line with our previous observations [3]. A similar pattern is observed in the uniaxial platform with uniaxial strains of 9.5% (U_9.5%_) and 3.5% (U_3.5%_) (Fig. S23), indicating a consistent response to both robotic and uniaxial mechanical stimuli across both platforms. These viability measurements reflect changes in metabolic activity and do not, on their own, distinguish between altered proliferation and other mechanically induced cellular responses.

To assess cell growth without interrupting culture, we explored the possibility of noninvasive monitoring under confocal microscopy directly through the thin transparent TPU membrane. A microscopy stage was developed to enable close contact between the cell membrane and the microscope objective (Fig. 4B and C). Images were successfully collected in situ from the same chambers on days 0, 2, 6, 10, and 14, under static, uniaxial, and robot stimulation, as shown in Fig. 4D. In contrast to previous mechanical stimulation platforms that involved cultivating cells on an elastomer membrane secured to a nontransparent base plate, and required cells to be fixed and stained before imaging, the noninvasive microscopy approach offers significant advantages: (a) It allows direct observation, avoiding the alterations in cell morphology that occur upon fixation; (b) it enables the monitoring of the same chamber over consecutive time points, reducing variability between samples; (c) it holds promise for future applications where the effects of mechanical dynamic forces on cell behavior can be continuously observed in real time.

Cell aspect ratio was computed as the ratio of the major to minor axes of fitted ellipses. Except for the U_3.5%_ group on day 10, all groups subjected to mechanical dynamic stimulation exhibit a significantly higher aspect ratio compared to the static group, indicating a more elongated cellular shape (Fig. 4E). On the robot platform, initially, the aspect ratio is higher for R_9.5%_ compared to R_3.5%_ on day 6, but later, R_3.5%_ exhibits a larger aspect ratio than R_9.5%_ on days 10 and 14. However, no significant differences are observed between U_3.5%_ and U_9.5%_. Moreover, in the groups subjected to mechanical dynamic stimulation, cells exhibit a more uniform orientation compared to those in static conditions (Fig. 4F and Fig. S24). Among the dynamically stimulated groups, R_3.5%_ shows more consistency in orientation than U_3.5%_, but overall, no significant differences are observed between the robot and uniaxial platforms. Previous studies have also shown that uniaxial stretching, used in tendon tissue engineering at various levels (2%, 5%, 10%, or 15% strain), can enhance the orientation of fibroblasts and MSCs [32–35]. These findings highlight the potential of our methods in advancing tissue engineering through improved understanding and application of dynamic mechanical stimuli.

### Cellular responses to mechanical stimuli: Gene and protein expression profile

**Fig. 5.**
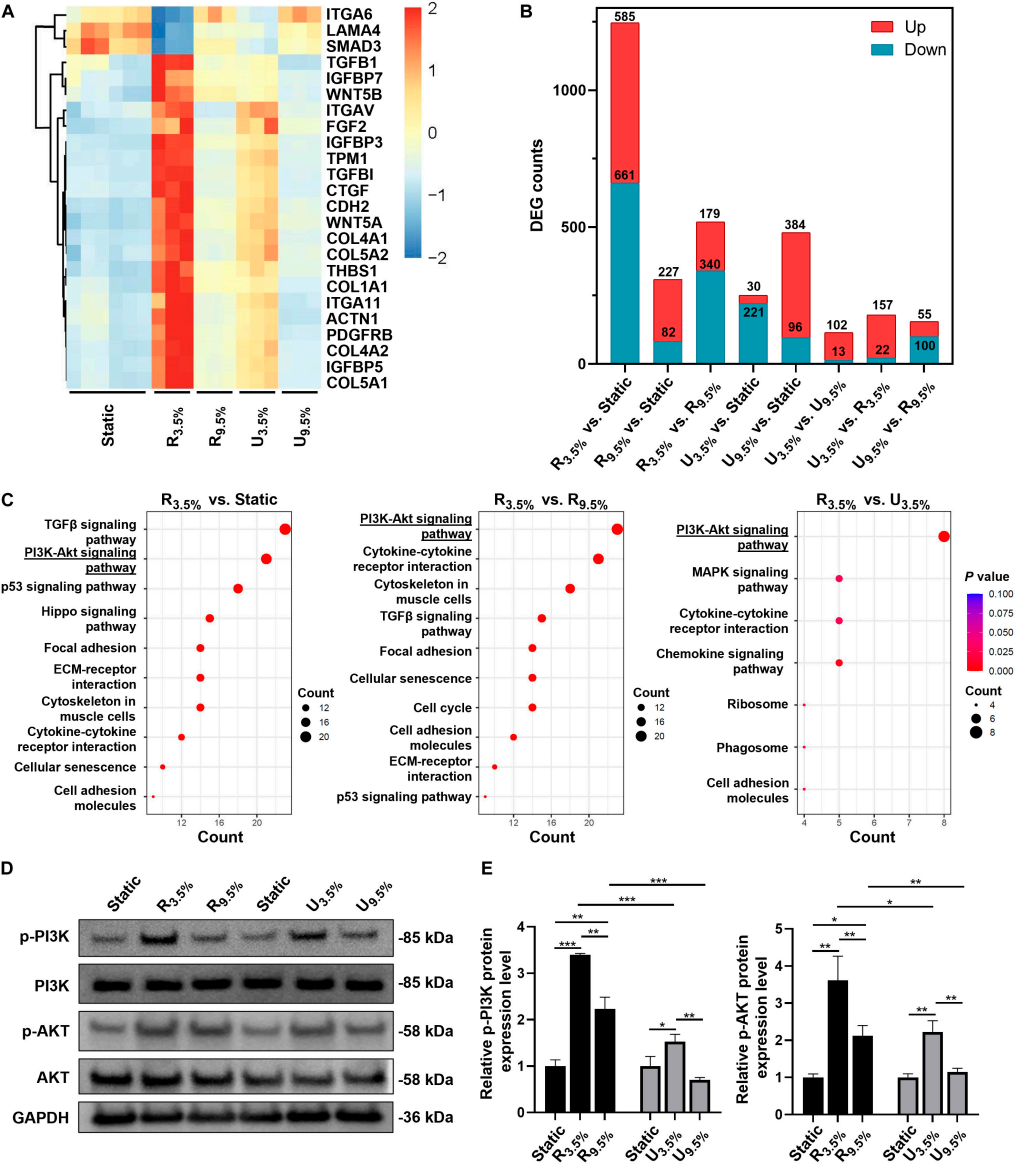
Cellular responses to mechanical stimuli: gene and protein expression profile. (A) Heatmap of differential gene expression in MSCs under static, uniaxial, and robot stimulation at different strains after 14 d. (B) Histogram showing the number of genes significantly up-regulated or down-regulated under each condition. (C) KEGG pathway analysis highlighting significant pathways across different groups. (D and E) Western blot and its quantitative analysis results for p-PI3K [p-PI3K/PI3K/glyceraldehyde-3-phosphate dehydrogenase (GAPDH)] and p-Akt (p-Akt/Akt/GAPDH), showing protein expression changes under static, uniaxial, and robot stimulation at different strains. Data are presented as mean ± SD (*n* = 3). Statistical significance was determined using 2-way ANOVA (*P < 0.05; **P < 0.01; ***P < 0.001).

The transcriptome of MSCs on day 14 was analyzed using bulk RNA sequencing. Sample quality control demonstrated low error rates, meeting the standards for further analysis (Table S1). The principal components analysis indicates clear differences between groups and good duplication within groups (Fig. S25). Following normalization, the differentially expressed genes were identified by setting thresholds at |log_2_(foldchange)| ≥ 1 and *P*_adj_ ≤ 0.05. The differentially expressed genes for each comparison are depicted in a heatmap (Fig. 5A and Fig. S26), histogram (Fig. 5B), and volcano plot (Fig. S27). The multiaxial robotic platform shows a greater number of differentially expressed genes between static, 25 N, and 50 N loading regimes when compared with the corresponding groups on the uniaxial control platform (Fig. 5B). The KEGG pathway analysis and GO pathway enrichment analysis identify enriched pathways and biological processes related to the differentially expressed genes (Fig. 5C and Figs. S28 and S29). Notably, the PI3K/Akt signaling pathway, associated with mechanical stimuli, is enriched on the robot platform for comparisons between R_3.5%_ versus static and R_9.5%_ versus R_3.5%_, with the uniaxial platform showing significance only in U_9.5%_ versus U_3.5%_ comparison (Fig. 5C).

Protein expression levels, validated through Western blot analysis, reveal that both platforms exhibit the highest levels of p-PI3K/PI3K and p-Akt/Akt under the strain of 3.5%, indicating the highest activation of the PI3K/Akt pathway (Fig. 5D to F). Moreover, the extent of change across all conditions is notably greater in the multiaxial robotic platform compared to the uniaxial platform. The comparative ratios of expression changes between conditions demonstrate this difference: For p-PI3K/PI3K, the ratios are 3.4:1.5 (R_3.5%_ to U_3.5%_) and 2.2:0.7 (R_9.5%_ to U_9.5%_); for p-Akt/Akt, the ratios are 3.6:2.2 (R_3.5%_ to U_3.5%_) and 2.1:1.1 (R_9.5%_ to U_9.5%_). These findings are consistent with previous reports highlighting the essential role of the PI3K/Akt pathway in regulating tenogenic differentiation and tendon formation as a downstream signaling mechanism of FAK in response to various mechanical stresses such as tension and compression [36–38].

## Discussion

Equipping flexible bioreactor chambers with soft sensors has enabled the precise application and monitoring of mechanical dynamic stimulation on growing cells with both the multiaxial robotic and uniaxial platforms. We have also successfully monitored cell growth noninvasively by performing confocal microscopy directly through the thin transparent TPU membrane. Our experiments have identified distinct transcriptomic and protein-level responses under matched peak tensile strain conditions, with multiaxial stimulation inducing greater changes in mechanosensitive signaling pathways compared with uniaxial loading. Remarkably, the integrated strain sensor verified that the applied strain magnitudes were comparable between platforms. Therefore, the distinct biological responses are likely associated with differences in mechanical load delivery mode rather than strain magnitude alone.

Under mechanical stimulation, a similar decrease in cell viability is noted in both platforms, particularly at 3.5% strain. However, this decrease should not necessarily be interpreted solely as evidence of acute cytotoxic damage. Although metabolic activity declined, microscopic observations and pathway activation suggest that this reduction may reflect a transition from proliferation to differentiation rather than cytotoxicity, in agreement with previous mechanobiology studies [39]. Consistent with this interpretation, the R_3.5%_ stimulation shows the highest activation of the PI3K/Akt pathway, which is widely implicated in mechanically regulated tenogenic signaling.

The distinct transcriptional and protein responses observed under multiaxial stimulation may be attributed to differences in how forces are transmitted at the cellular scale. While uniaxial stretch predominantly applies force along a single axis, multiaxial deformation can generate heterogeneous strain fields, introducing combinations of tensile, compressive, and shear components across the tendon scaffold. These complex mechanical cues are likely sensed through integrin–focal adhesion complexes and transmitted via the actin cytoskeleton, promoting focal adhesion turnover and nuclear deformation [40]. Such mechano-nuclear coupling may enhance chromatin accessibility and amplify downstream pathways including the PI3K/Akt signaling axis. The observed differences in mechanosensitive signaling responses was therefore not attributed to increased strain magnitude, but to the directional complexity of strain experienced within the scaffold. Although only a single joint axis was actuated, the compliant bioreactor chamber permitted subtle off-axis deformation within the scaffold, thereby creating a multiaxial strain environment at the tissue scale.

Various tissue engineering strategies have been deployed to mimic the complex mechanical environments found in vivo [41], utilizing cutting-edge technologies such as shear flow [42], physical membrane indentation [43], magnetic tweezer manipulations [44], axial global cellular stretch [45], and both uniaxial and multiaxial elastomer membrane stretch systems [46]. However, many in vitro models still apply strain isotropically and fail to capture the directionality of mechanical cues experienced by native tendon tissue. Although tensile strain remains the primary force acting on supraspinatus tendons, in vivo studies have reported heterogeneous intratendinous strain patterns and potential shearing between tendon layers during arm elevation. By employing a humanoid robotic shoulder platform, this study introduces a loading environment that more closely approximates the mechanical complexity of the human shoulder. This innovative approach allows for the application of torsion, compressive, and tensile stresses through a non-load-bearing membrane. These advancements have led to significant improvements in cellular orientation and pronounced activation of mechanosensitive signaling pathways, particularly PI3K/Akt, compared to the uniaxial platform.

Our approach has demonstrated promising results; however, several limitations must be addressed in future work. The present study was intentionally designed to interrogate early mechanotransductive and cellular responses to complex loading, rather than long-term tissue maturation or construct-level functionality. First, although the robotic platform is capable of 3 kinematic DOF, only a single DOF was employed in this study, which, while relevant to supraspinatus loading, does not fully reproduce the complex motion patterns present in daily and skilled activities. Future work will incorporate additional joint rotations to more accurately simulate physiological loading. Second, periodic media replacement does not adequately mimic the dynamic nutrient and waste exchange seen in vivo. A continuous perfusion system will be integrated to better simulate the native tissue environment and address potential challenges like continuous media supply and hypoxia during long-term culture. Third, the culture period was limited to 14 d. Extending the culture duration beyond 1 month will be crucial for assessing sustained mechanobiological adaptation. Fourth, the use of MSCs and primary fibroblasts does not capture the full cellular heterogeneity of tendon tissue. Inclusion of tendon-derived cells, endothelial populations, and vascular elements will be important to improve physiological relevance. Fifth, no functional assessment of the constructs was conducted at the endpoint. In addition to the absence of histological evaluation for tendon-like matrix deposition, such as collagen type I or tenomodulin, mechanical testing and analysis of ECM organization will be required in future studies to determine whether multiaxial loading promotes construct-level maturation. In addition, integrating sensors to monitor key nutrients, metabolites, and environmental factors such as oxygen levels, along with developing computational models, will provide a deeper understanding of the cellular environment and contribute to more effective tendon repair strategies. While passive shear or torsional deformation may occur within the bioreactor chamber, these components were not quantified in the present study. Finally, although the MEF sensor exhibits reliable performance, its measured strain does not represent the strain of the scaffold or embedded cells, as it is not perfectly attached to the matrix. Future work will address these limitations through full integration of the sensor and matrix.

## Data Availability

The data and materials used to support the findings of this study are available from the corresponding author upon reasonable request.

## Supplementary Materials

Supplementary 1 Graphical Abstract Figs. S1 to S30 Tables S1 to S3
