# Supplementary material for: Humanoid Robotic Loading Enhances Mechanotransduction in Tendon Tissue Engineering
Source: Cyborg Bionic Syst. 2026 Mar 24;7:0542. doi: 10.34133/cbsystems.0542 (PMC13009534; doi:10.34133/cbsystems.0542)
Supplement: Supplementary 1 — Graphical Abstract Figs. S1 to S30 Tables S1 to S3 [file cbsystems.0542.f1.zip › Revised Supporting Information.docx]

Supporting Information

**Humanoid robotic loading enhances mechanotransduction in tendon tissue engineering**


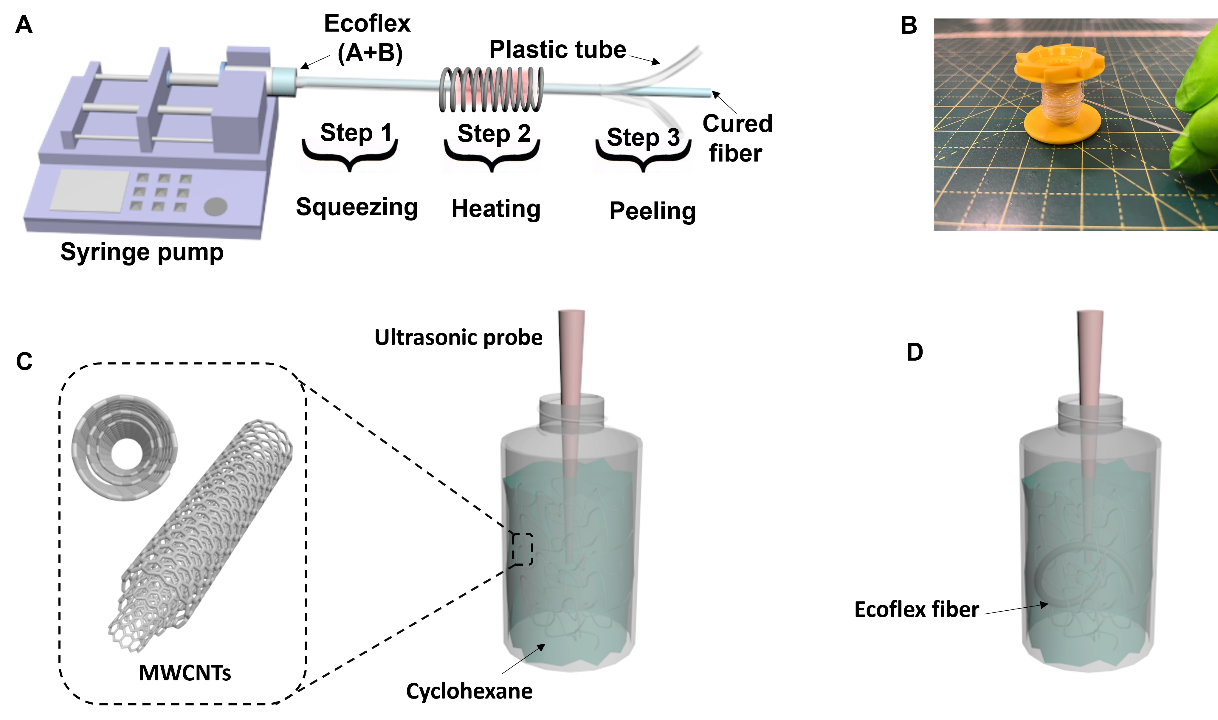


**Figure S1.** Preparation of Ecoflex fibre and coating it with MWCNTs. A) Preparation of Ecoflex fibre through extruding freshly prepared Ecoflex into plastic tubing, heating, and then peeling the tubing off. B) The image of a roll of Ecoflex fibre. C) Ultrasonic treatment of MWCNTs in cyclohexane to disperse the MWCNTs before Ecoflex fibre coating. D) Coating the Ecoflex fibre in the prepared dispersion with ultrasonic treatment and then taking the fibre out to complete the assembly.


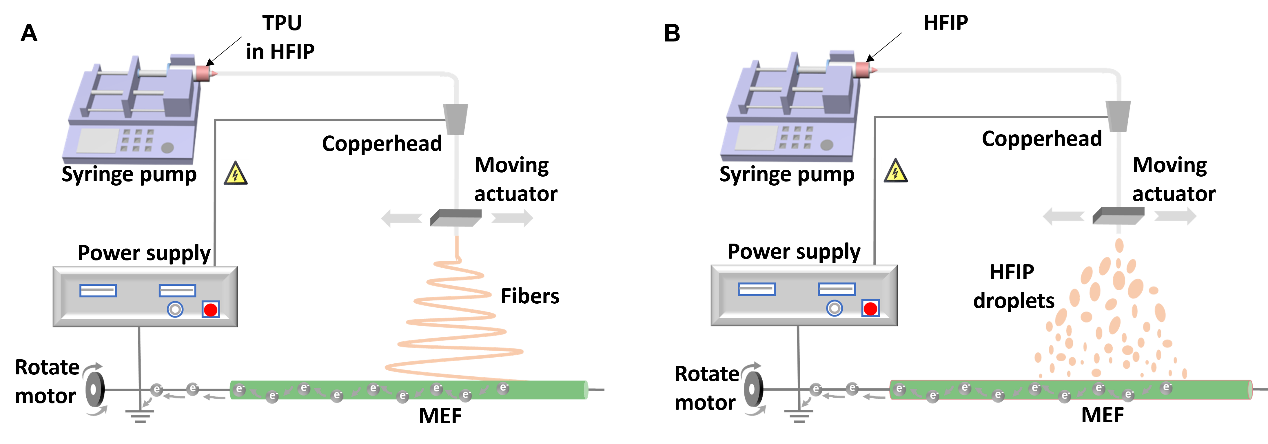
**Figure S2.** Encapsulating MEF by electrospinning and electrospray aims to avoid electrical contact with cells during mechanical stimulation in strain sensing. A) Applying TPU fibres onto the MEF through an electrospinning technique, with the MEF serving as a collector that rotates along its axis back and forth. B) Implementing electrospray of 1,1,1,3,3,3-Hexafluoroisopropanol (HFIP) on the electrospun fibers with the same experimental setup.


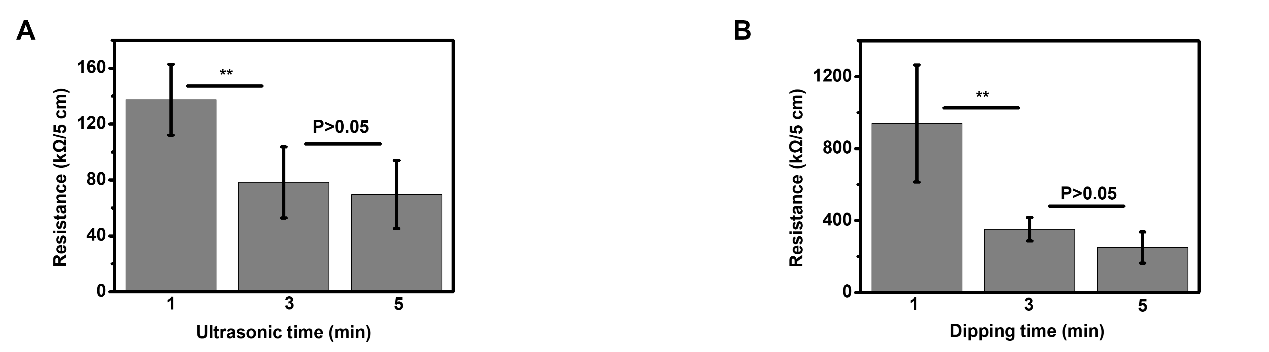


**Figure S3.** Resistance of the Ecoflex fibres after being modified by MWCNTs with different treatment time. A) Resistance of the fibre after assembling MWCNTs with ultrasonic treatment. B) Resistance of the fibre after assembling MWCNTs with dipping coating. The fibres demonstrate a noticeable reduction in resistance as the ultrasonic treatment duration increases. Specifically, the resistance values remain relatively consistent for fibers treated for 3 and 5 min. Interestingly, a similar trend is observed when modifying the fibre solely through dipping coating, albeit with higher resistance compared to the ultrasonic-treated group.


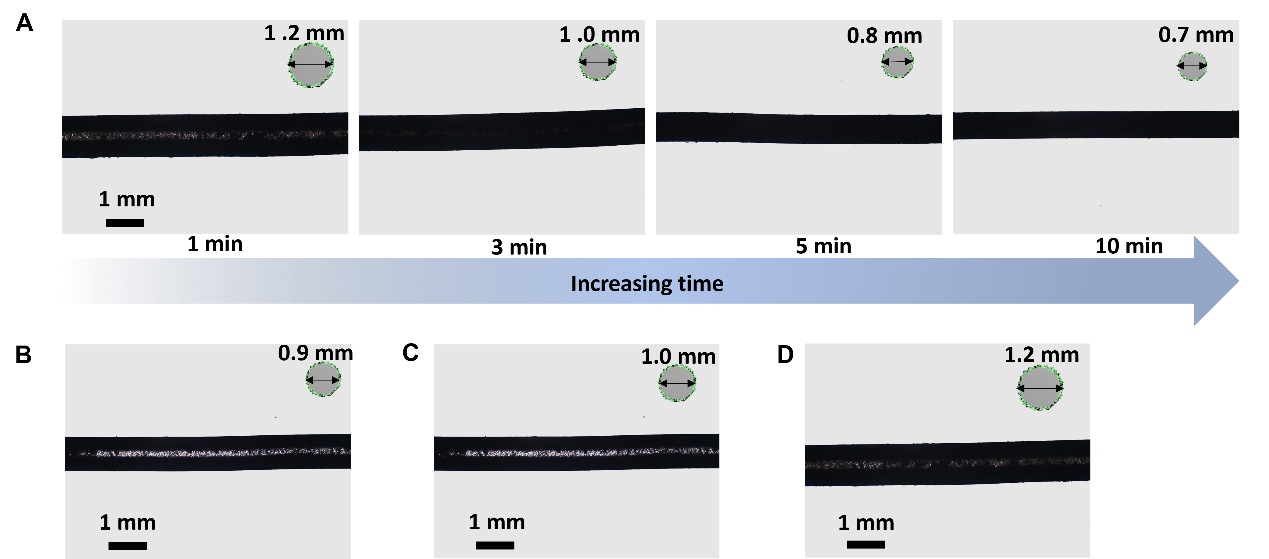


**Figure S4.** Optical images and corresponding diameters (top right inserts). A) Optical images and corresponding diameters of the MCF with ultrasonics for 3 min at specific times (1, 3, 5, 10 min) after removal from the dispersion. Optical images and corresponding diameters of the MCFs with B) dip coating for 3 min at 1 min after removal from the dispersion, C) ultrasonics for 1 min at 1 min after removal from the dispersion, and D) ultrasonics for 5 min at 1 min after removal from the dispersion.

The swollen fibres gradually recover over time after being removed from the dispersion (Figure S4A). The fibre subjected to direct dip coating for an equivalent duration (Figure S4B) displays a smaller diameter (after removal for 1 min) compared to the fibre coated using ultrasonics (Figure S4C). However, the diameters of the fibres treated with ultrasonics for 5 min (Figure S4D) and 3 min (Figure S4A) converge to the same diameter of 1.2 mm after 3 min.


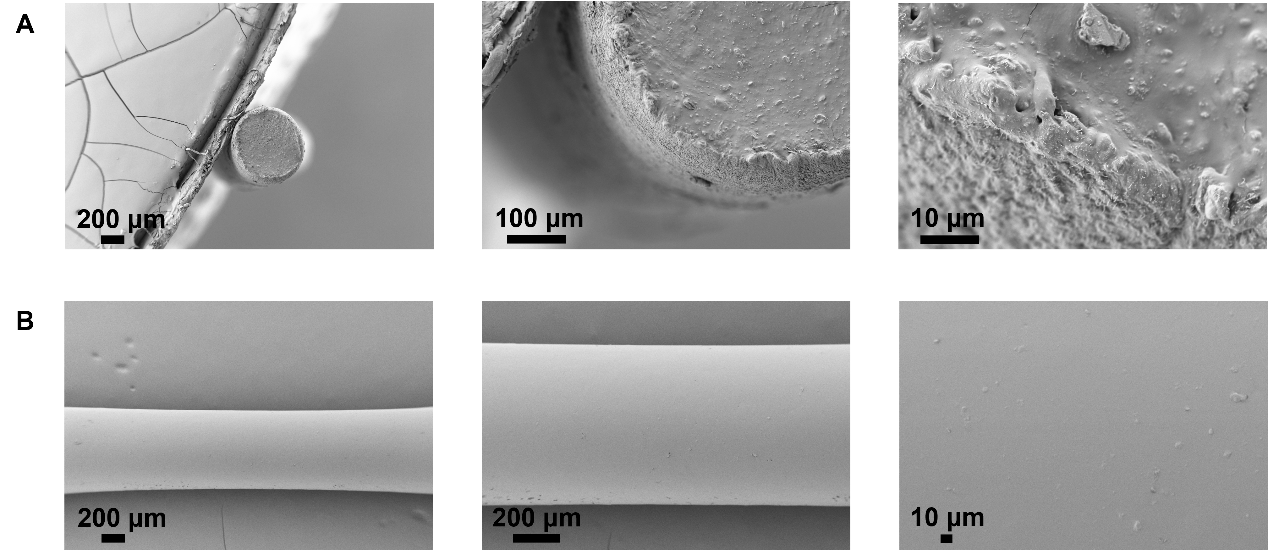


**Figure S5.** Morphology of the MEF and Ecoflex fibre. A) Section morphology of the MEF with ultrasonics for 3 min, showing the MWCNTs only on the fibre surface. B) Surface morphology of the Ecoflex fibre with ultrasonic treatment for 3 min in pure cyclohexane without MWCNTs, showing there are no wrinkles on the surface.


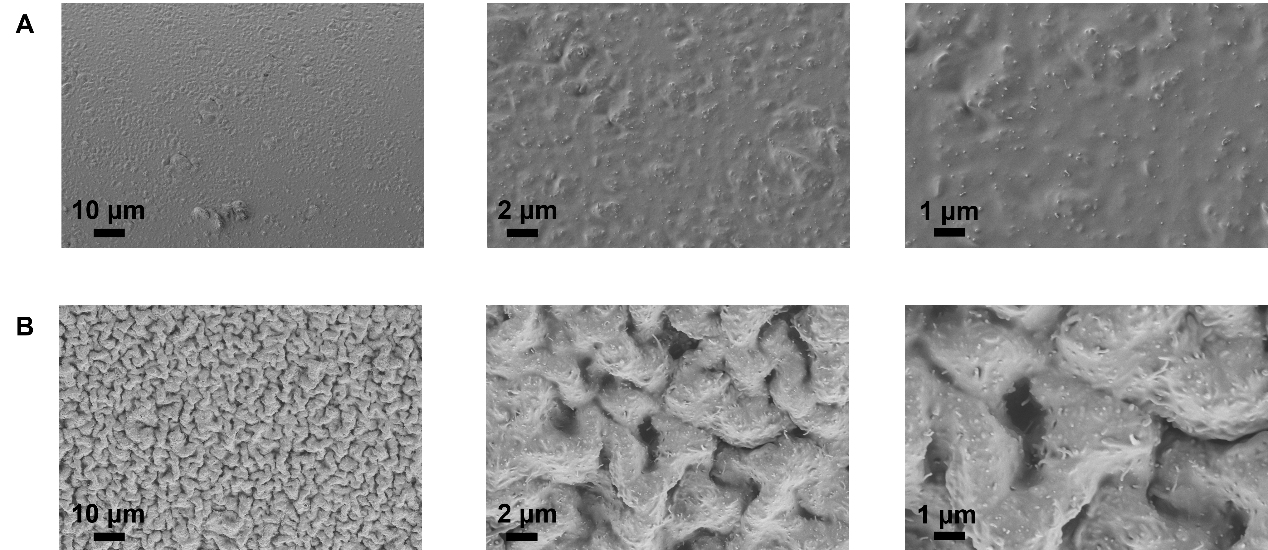


**Figure S6.** Surface morphology of the MEFs with ultrasonics for A) 1 min and B) 5 mins.


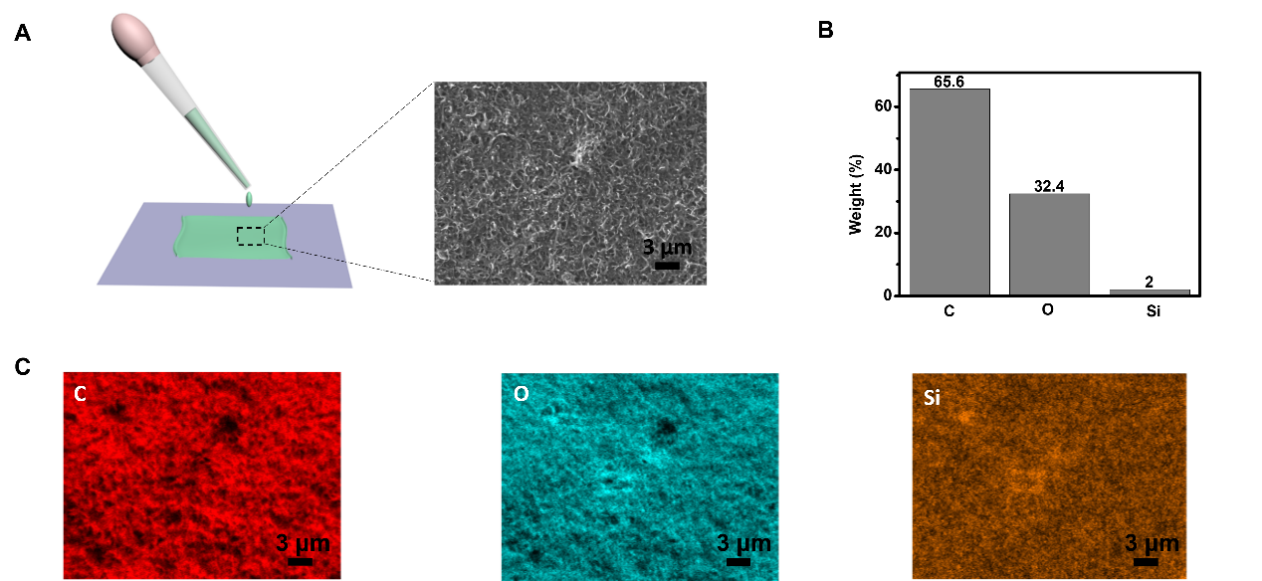


**Figure S7.** Element analysis of dry MWCNTs dispersion with cyclohexene evaporated after Eocflex fibre modification. A) Sample prepare illustration and its morphology. B) Element content and percentage of the sample. C) Element distribution of the sample.


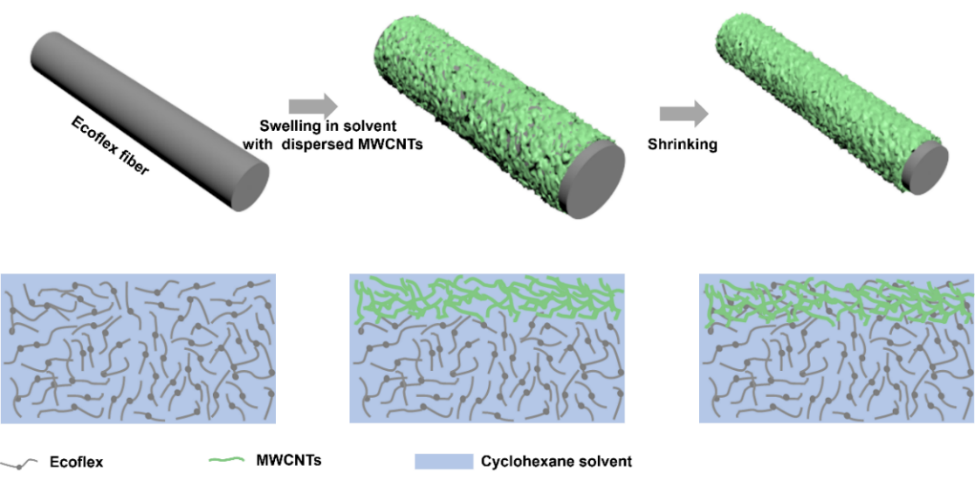


**Figure S8.** Proposed mechanism of MWCNTs assembling on Ecoflex fibre.


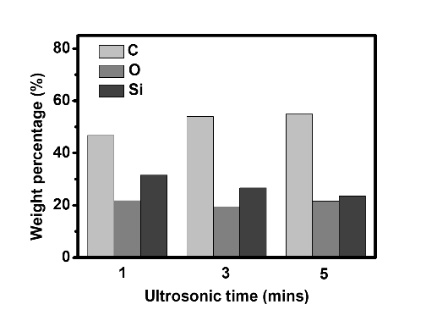


**Figure S9.** Surface element content of the MEFs with ultrasonic treatment for 1, 3 and 5 min.

To gain insight into the assembly mechanism, we extracted the used dispersion onto a conductive carbon tape (Figure S7A) and waited for the evaporation of cyclohexane before conducting an elemental analysis using energy-dispersive X-ray spectroscopy (EDS). The analysis revealed a certain content of Si (i.e., 2% in weight) in the composite, indicating the incorporation of Ecoflex into the dispersion during assembly (Figure S7B, C). Based on this, we proposed an assembly mechanism (Figure S8). The Ecoflex swelling in cyclohexene is the gradual diffusion process of Ecoflex, and simultaneously, there is a layer of MWCNTs attached onto the Ecoflex surface. After being taken out from the dispersion, the Ecoflex gradually recovered with cyclohexene evaporating, thus forming wrinkles on the surface due to the MWCNTs. This proposed mechanism explains why ultrasonic treatment enhances conductivity and why the MEF with ultrasonic for 3 min reaches the equally minimum resistance, as ultrasonic treatment intensifies fiber swelling, reaching its maximum at 3 min (Figure S4A). The larger swelling surface results in more MWCNTs being attached to the Ecoflex, ultimately leading to improved conductivity. The increased content of the MWCNTs is supported by the surface EDS analysis as well (Figure S9). It is important to note that the wrinkles contain both MWCNTs and Ecoflex rather than pure MWCNTs from the EDS analysis, and it is also supported by the fibre surface morphology (Figure S6).


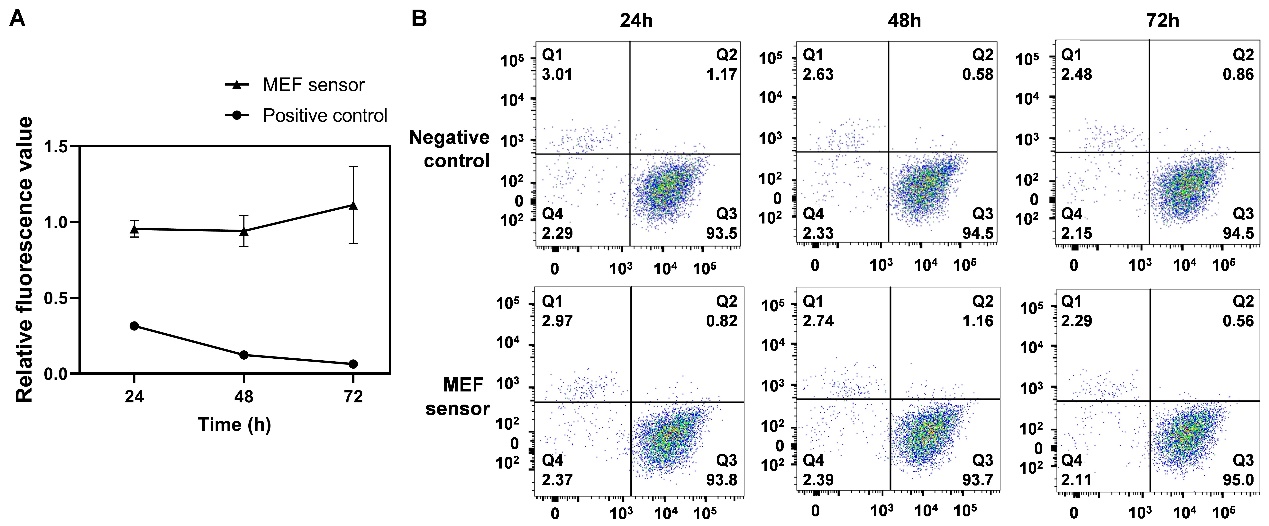


**Figure S10.** The biocompatibility of the MET sensor. A) PrestoBlue cell viability assay shows the relative cell viability, with the y-axis representing fluorescence intensity compared to the negative control. Data are presented as mean ± SD (n=3). B) Flow cytometry assesses the cell viability through live/dead staining. The y-axis in the Q3 quadrant displays the percentage of live cells.


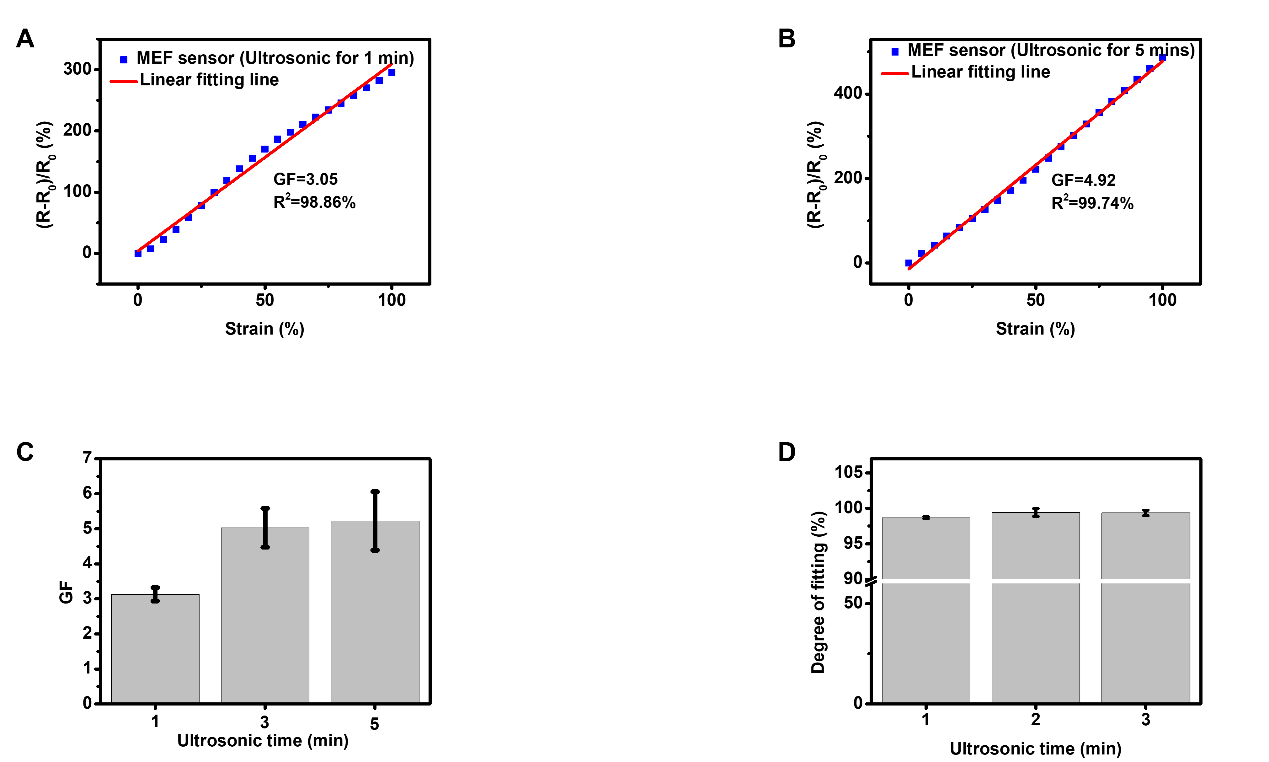


**Figure S11.** Sensing performance summary of MEF sensors with ultrasonic treatment for different time. A) Typical calibration and linear fitting of the MEF sensor with ultrasonic treatment for 1 min. B) Typical calibration and linear fitting of the MEF sensor with ultrasonic treatment for 5 mins. C) GF summary of repeat MEF sensors. D) Fitting degree summary of repeat MEF sensors. Data are presented as mean ± SD (n=3).


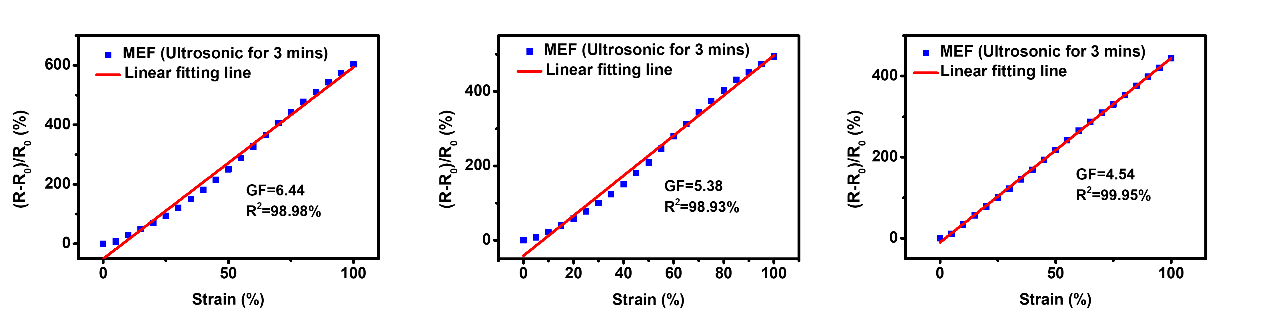


**Figure S12.** Calibration and linear fitting of MEF with ultrasonics for 3 min, left to right referring to different samples. The TPU encapsulation does not significantly affect the sensor sensitivity by comparing to the calibration data of the MEF sensor with ultrasonics for 3 min (Figure S10C).


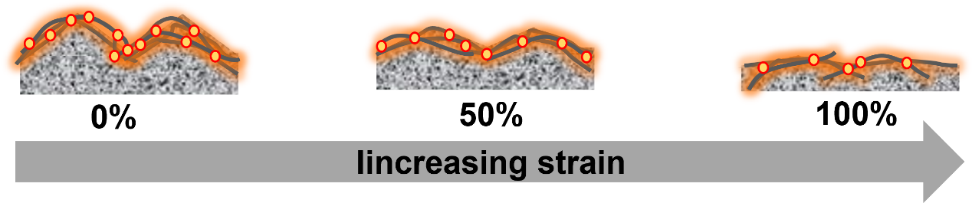


**Figure S13.** Illustration of the surface of the MEF upon stretching, showing the sensing mechanism because the separation of the surface MWCNTs increases the resistance.


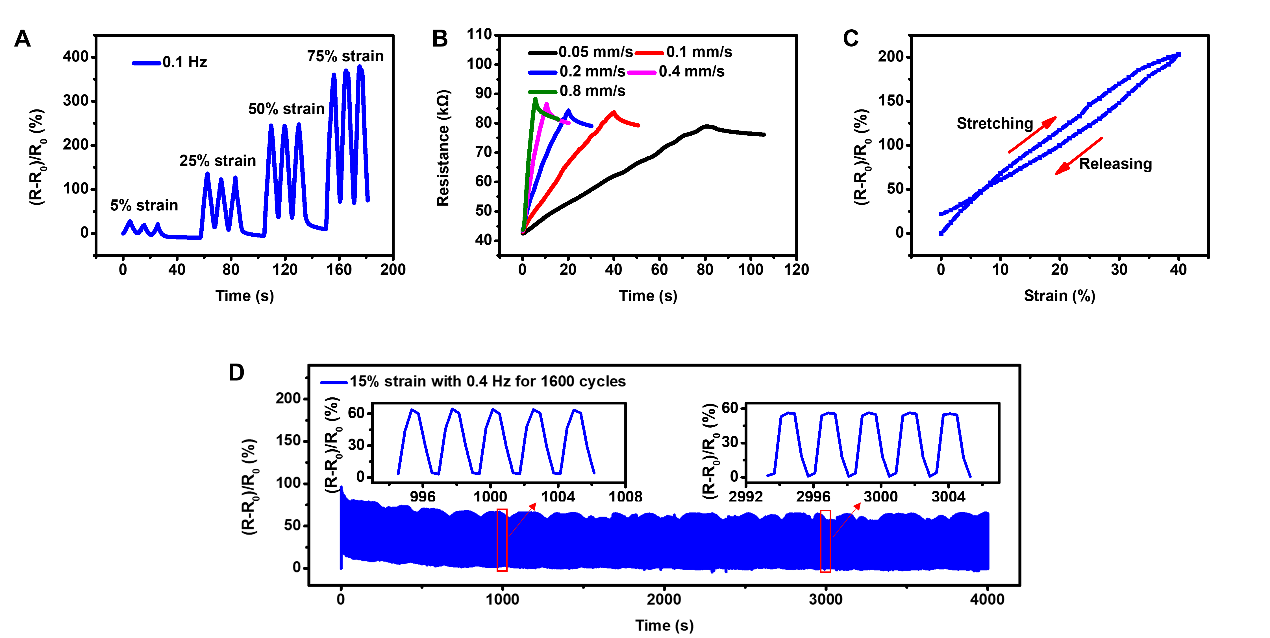


**Figure S14.** Electromechanical performance of the MEF sensor. A) Relative resistance changes with repeat frequency of 0.1 Hz under various strains to show its dynamic stability. B) Resistance signals by applying a strain of 20% and maintaining the deformation, with various stretching speed. C) Relative resistance changes upon a loading-unloading strain from 0% to 40%, showing the sensor hysteresis. D) Electrical signals at an applied cyclic strain of 15% with the frequency of 0.4 Hz for 1600 cycles, showing the high durability.


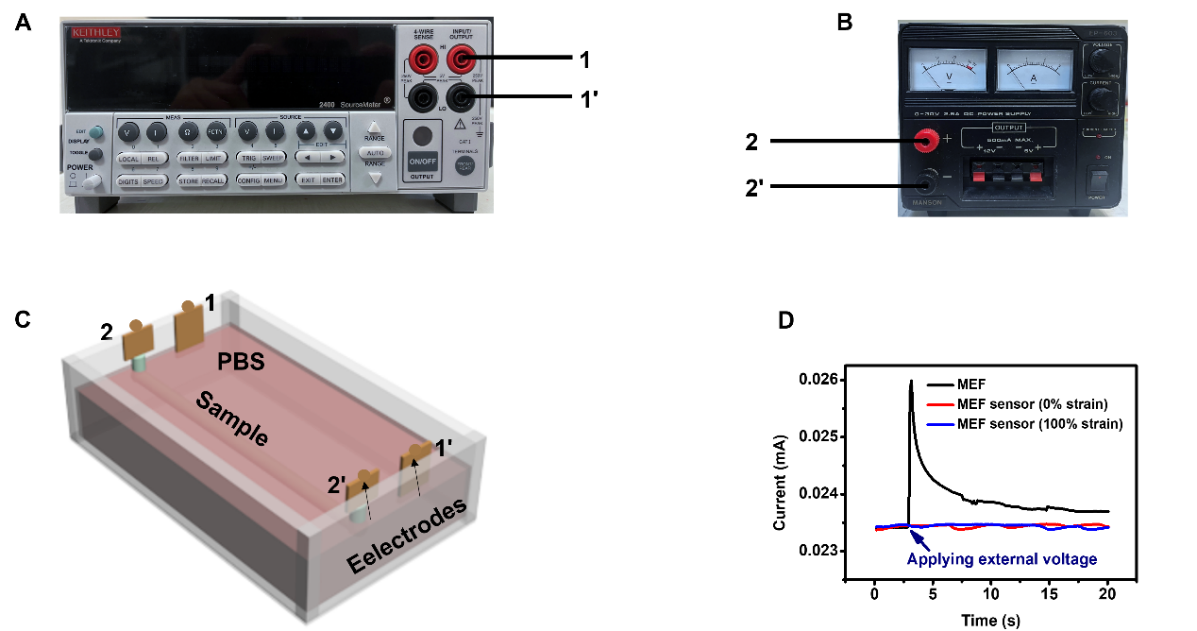


**Figure S15.** Experimental setup and electrical outputs to confirm TPU encapsulation preventing current leaks. A) A multimeter to detect the current in PBS. B) A power supplier to apply current to samples. C) Illustration of the evaluation device, where the middle part of testing sample is immersed into PBS with current getting through, and multimeter is connected the PBS to detect if there is current. D) Electrical outputs, showing that the TPU encapsulation prevents the current leakage even under the strain of 100%.


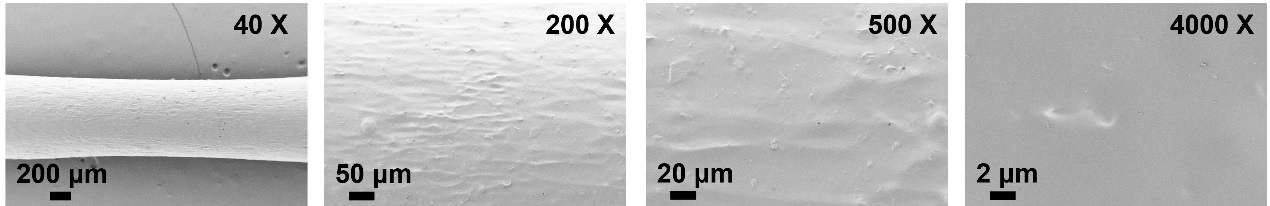


**Figure S16.** The different-multiple surface morphology of the MEF sensor under the strain of 100%.


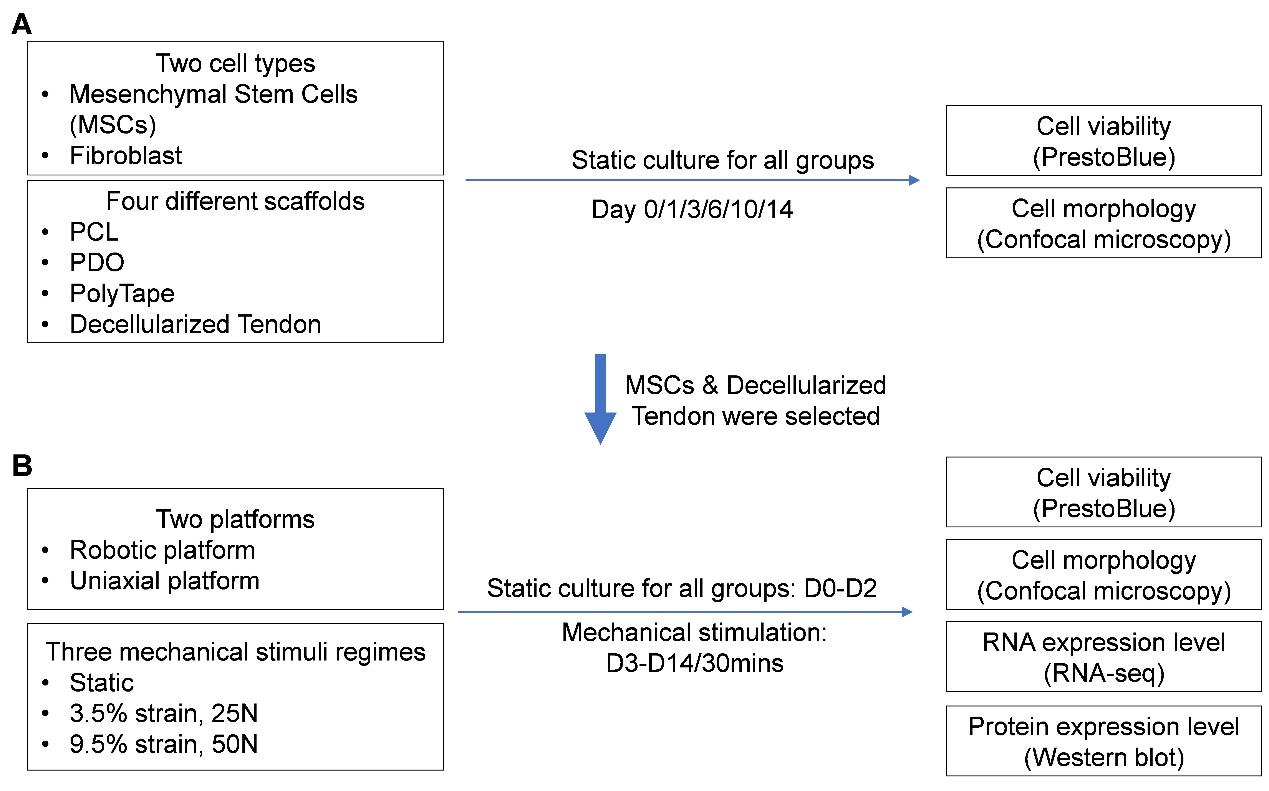


**Figure S17.** Schematic representation of cellular experiment workflow. The experiment encompasses two primary evaluations. A) MSCs and fibroblast responses on scaffolds—assessing MSCs and fibroblasts grown on PCL, PDO, PolyTape, and decellularised tendon scaffolds. Viability (PrestoBlue) and morphology (confocal microscopy) were analysed on day 0, 1, 3, 6, 10 and 14. B) Responses to mechanical stimuli—under static, 25 N, and 50 N force regimes on both multiaxial and uniaxial platforms. Evaluations included cell viability (PrestoBlue), morphology (confocal microscopy), and molecular profiling (RNA and protein expression levels).


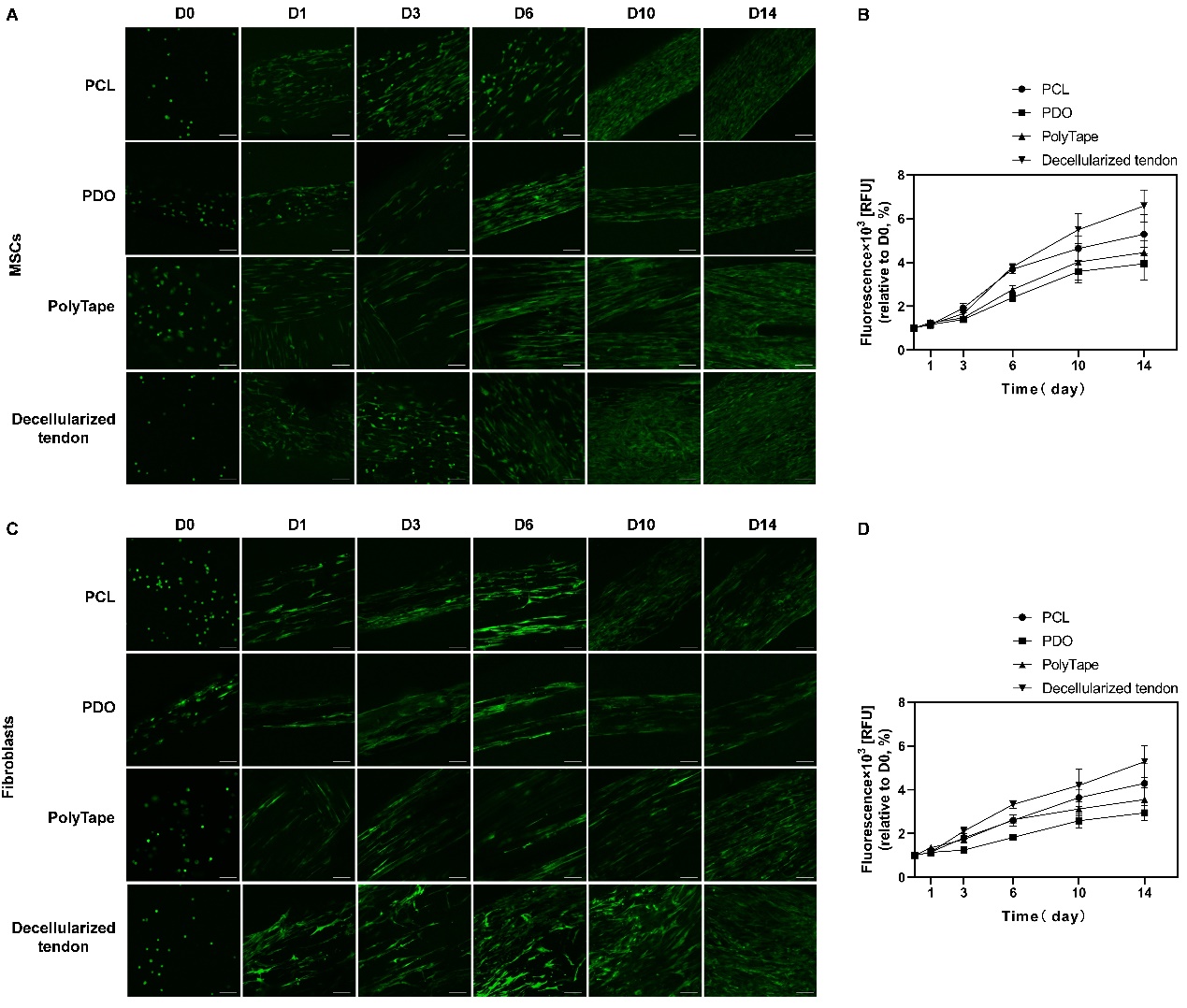
 **Figure S18**. Cell and scaffold selection. A) Confocal morphology observation of MSCs grown on PCL, PDO, PolyTape, and decellularised tendon scaffolds. Images were captured on days 0, 1, 3, 6, 10, and 14. Scale bar=100 µm; B) Cell viability assessment of MSCs: PrestoBlue assay results indicating the viability of MSCs on each scaffold across the designated time points; C) Confocal morphology observation of fibroblasts grown on PCL, PDO, PolyTape, and decellularised tendon. Images were captured at days 0, 1, 3, 6, 10, and 14. Scale bar=50 µm; D) Cell viability assessment of Fibroblasts: PrestoBlue assay results indicating the viability of fibroblasts on each scaffold across the designated time points. Data are presented as mean ± SD (n=3).


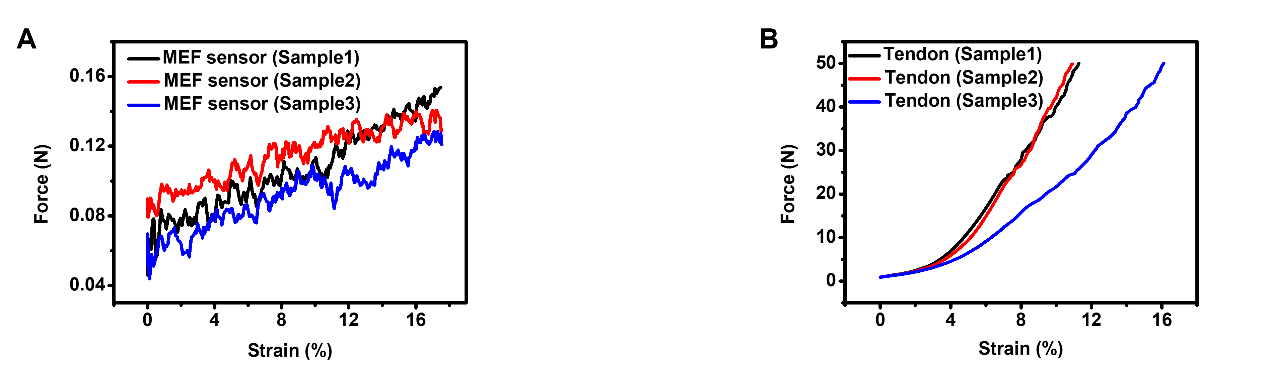


**Figure S19.** Mechanical performance of MEF sensors and decellularised tendons. A) Strain-force curves of 3 MEF sensors by stretching them to the strain of 17.5%. B) Strain-force curves of three decellularised tendons by stretching them to the maximum force of 50 N.


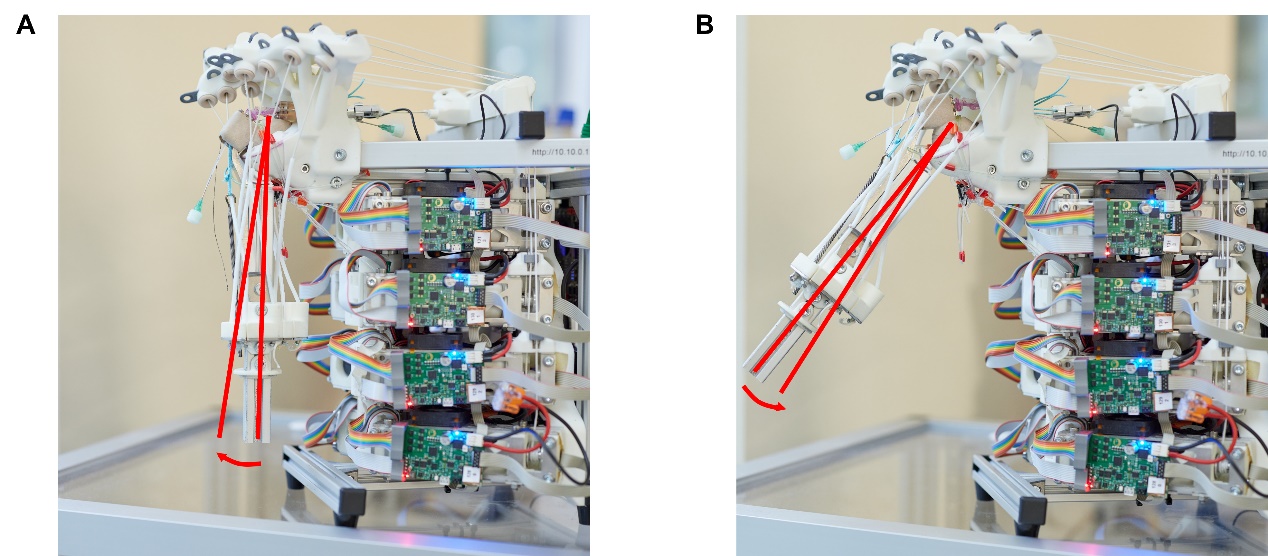


**Figure S20.** Images and illustrations of humanoid robot shoulder at different conditions. A) Illustration of the arm abduction to a certain degree from the original state, where the force strikes the first peak. B) Illustration of the arm adduction to a certain degree from maximum abduction, where the force strikes the second peak.


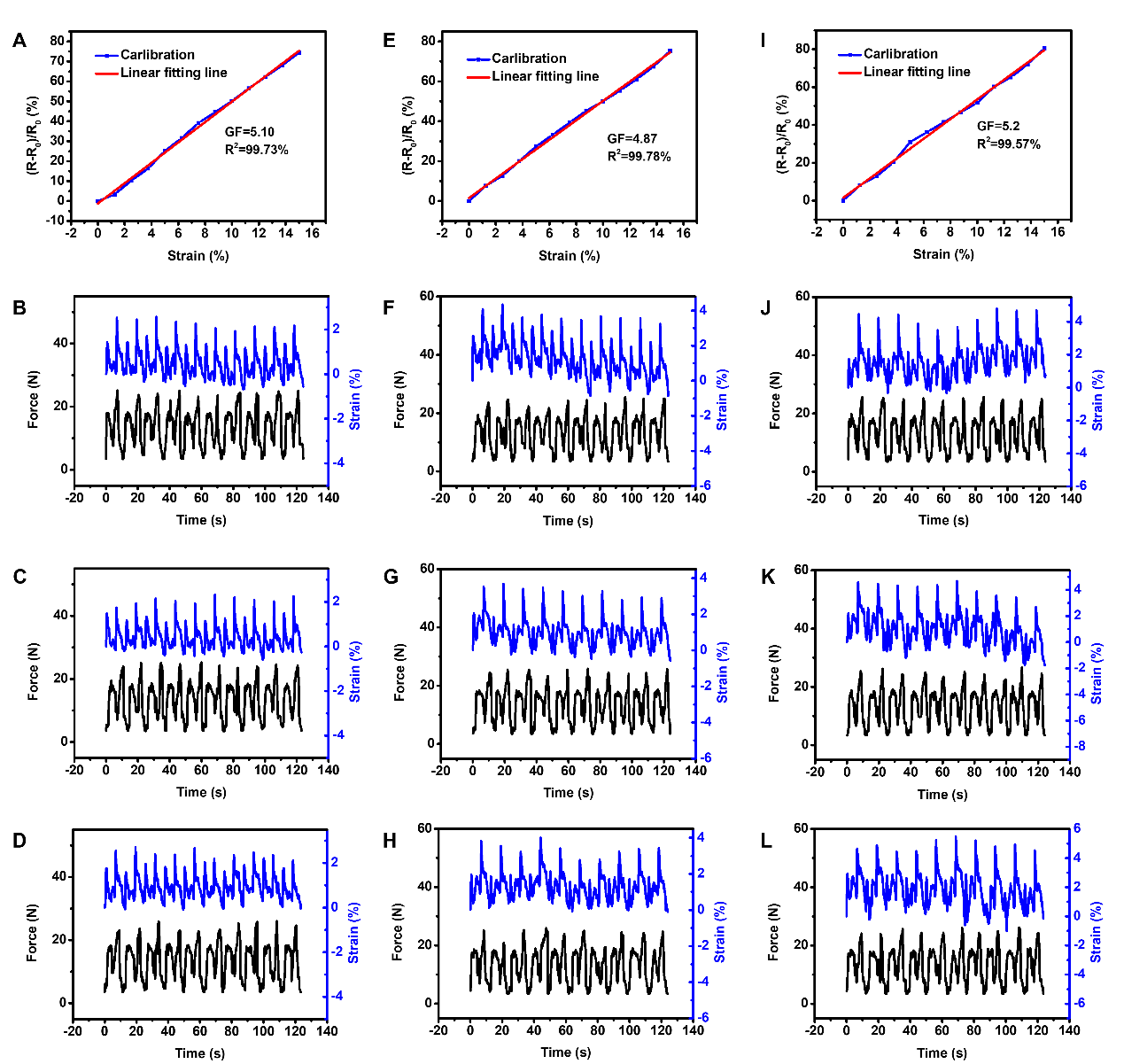
 **Figure S21.** Calibration of 3 MEF sensors and 10 cyclic force-strain curves under the stimulation load of 25 N at days 3, 8 and 14 after injecting cells into the bioreactor. A) Calibration of a MEF sensor and, B-D) the force-strain curves at days 3, 8 and 14, where the strain is calculated by the sensor signals. E) Calibration of a MEF sensor and F-H) the force-strain curves at day 3, 8 and 14, where the strain is calculated by the sensor signals. I) Calibration of a MEF sensor and J-L) the force-strain curves at days 3, 8 and 14, where the strain is calculated by the sensor signals.


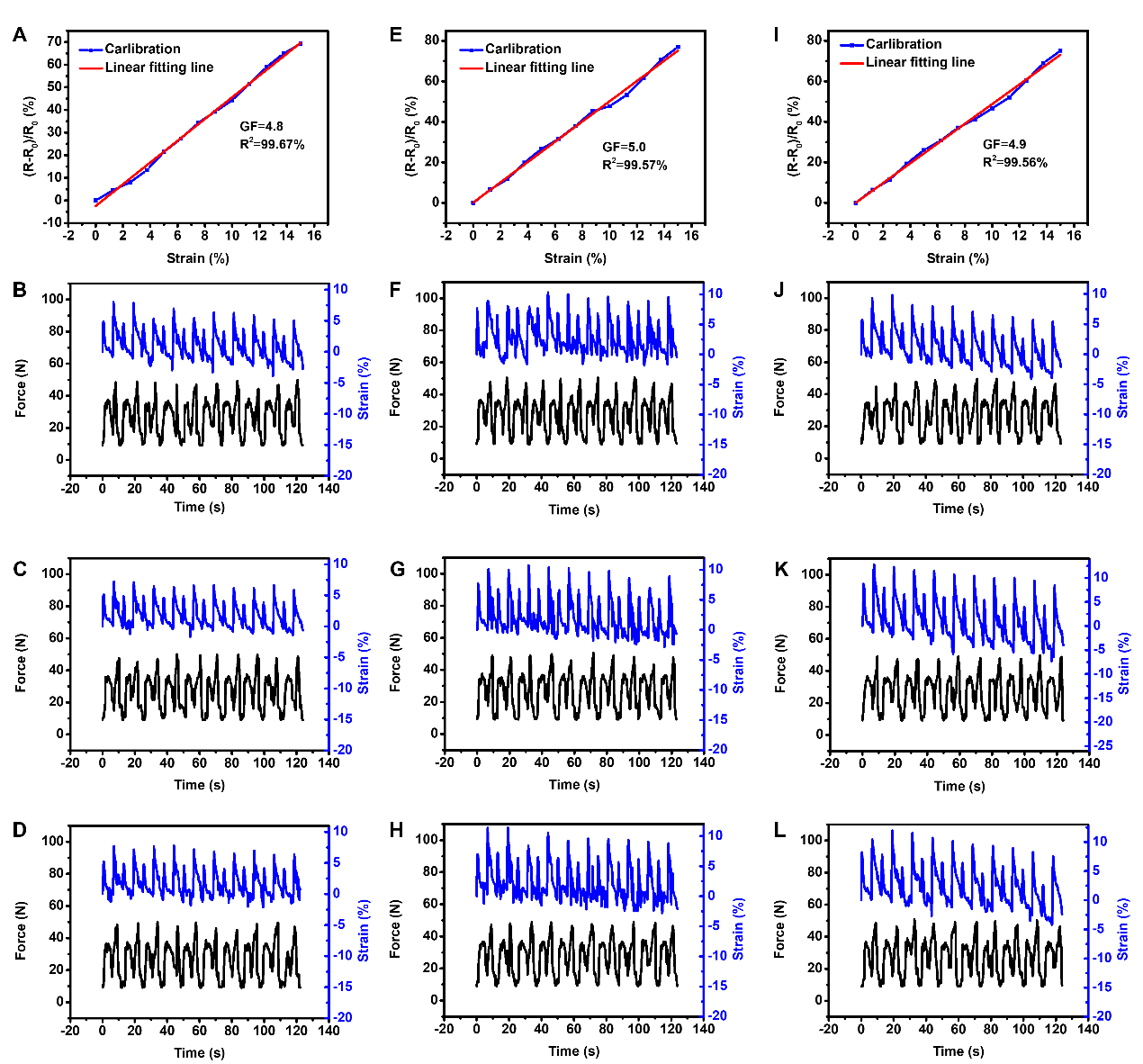


**Figure S22.** Calibration of three MEF sensors and 10 cyclic force-strain curves under the stimulation load of 50 N at days 3, 8 and 14 after injecting cells into the bioreactor. A) Calibration of a MEF sensor and B-D) the force-strain curves at days 3, 8 and 14, where the strain is calculated by the sensor signals. E) Calibration of a MEF sensor and F-H) the force-strain curves at days 3, 8 and 14, where the strain is calculated by the sensor signals. I) Calibration of a MEF sensor and J-L) the force-strain curves at days 3, 8 and 14, where the strain is calculated by the sensor signals.


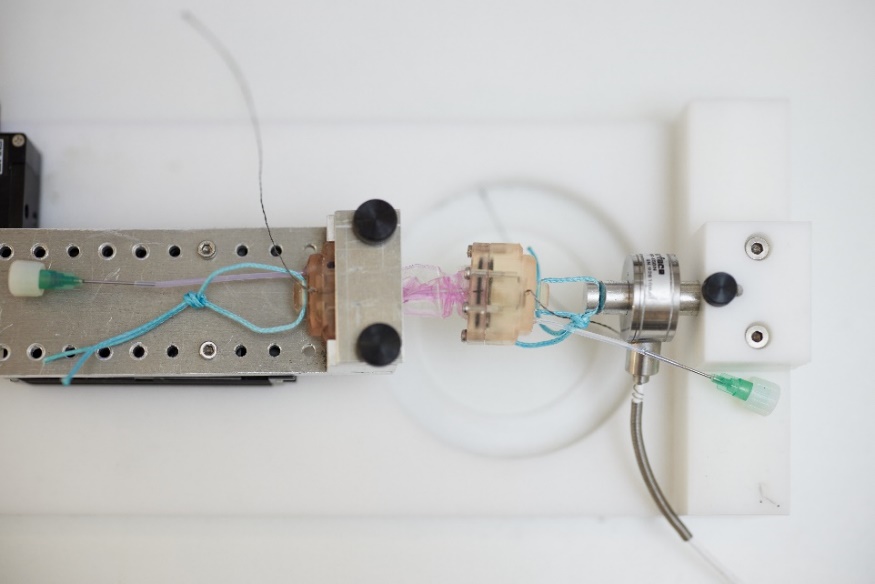


**Figure S23.** An image of the uniaxial platform that implements mechanical dynamic stimulation.


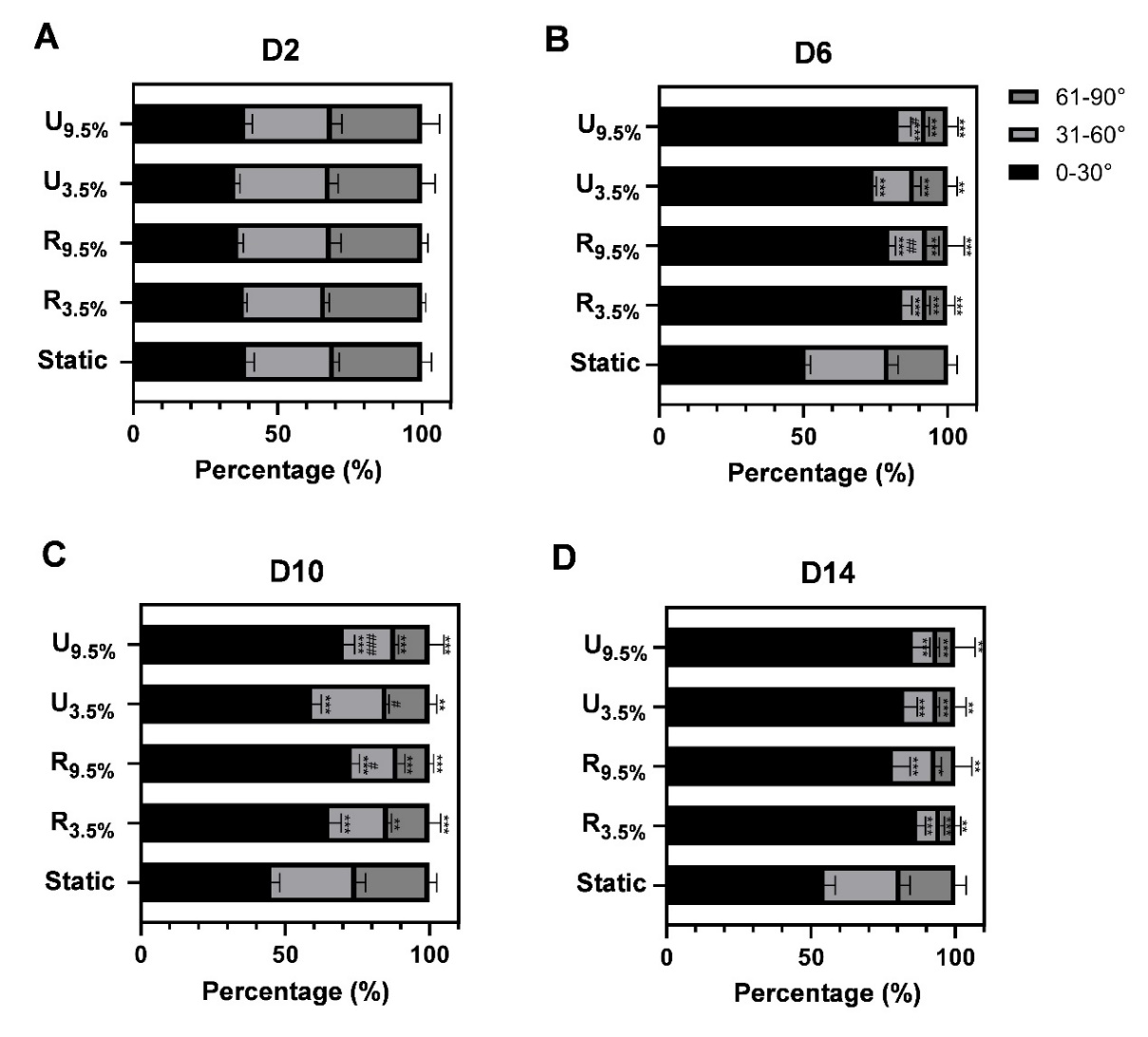


**Figure S24.** Analysis of cell orientation. A-D) The cell orientation at days 2, 6,10 and 14, where the orientation angles were divided into three groups: 0°-30°, 31°-60°, and 61°-90°. Data are presented as mean ± SD (n=3). Statistical significance was determined using two-way ANOVA (*, p < 0.05; **, p < 0.01; ***, p < 0.001).


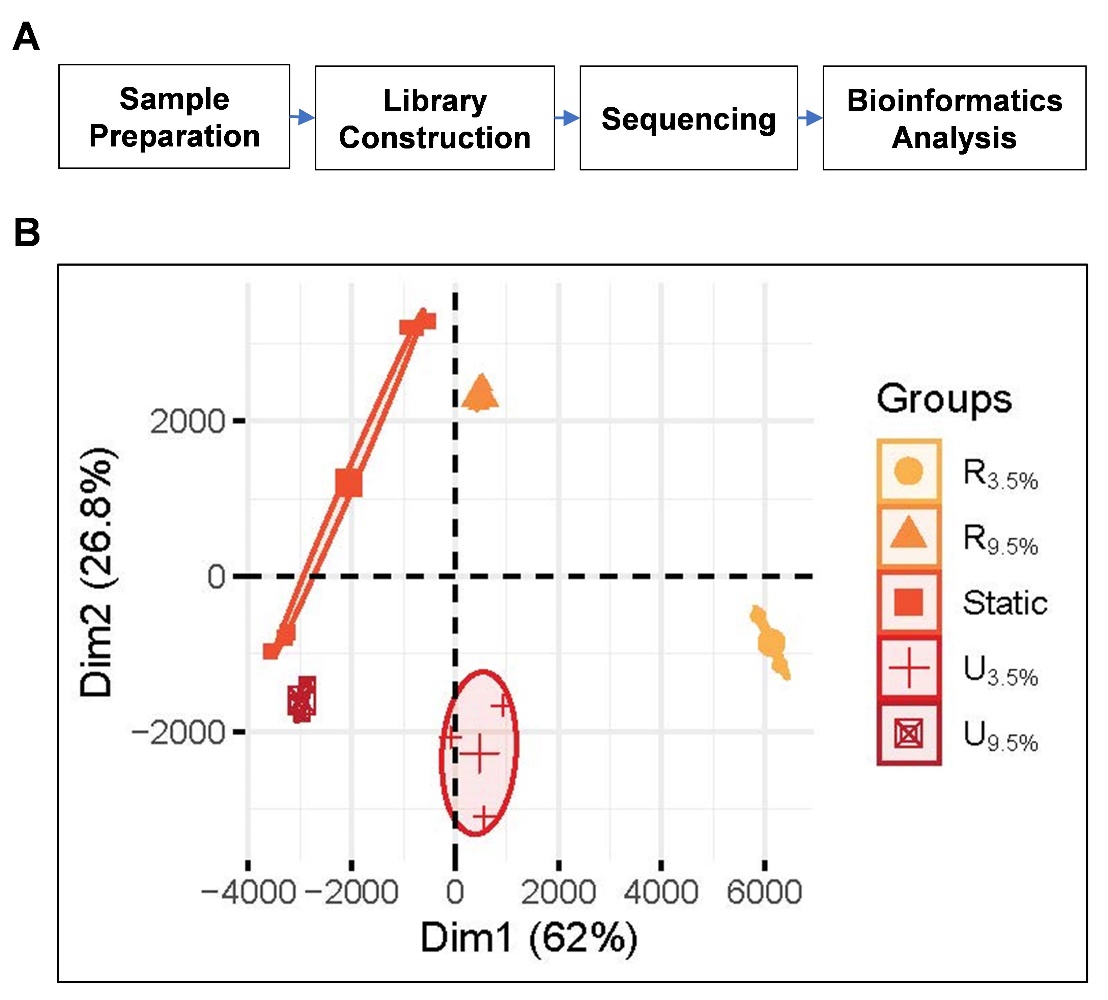


**Figure S25.** RNA-seq project workflow and quality control. A) RNA-seq project workflow outlines the steps involved in RNA-seq experiment from sample preparation to data analysis. B) Principal component analysis results show distinct clusters for different groups and tight groupings for samples within the same group based on gene expression values.


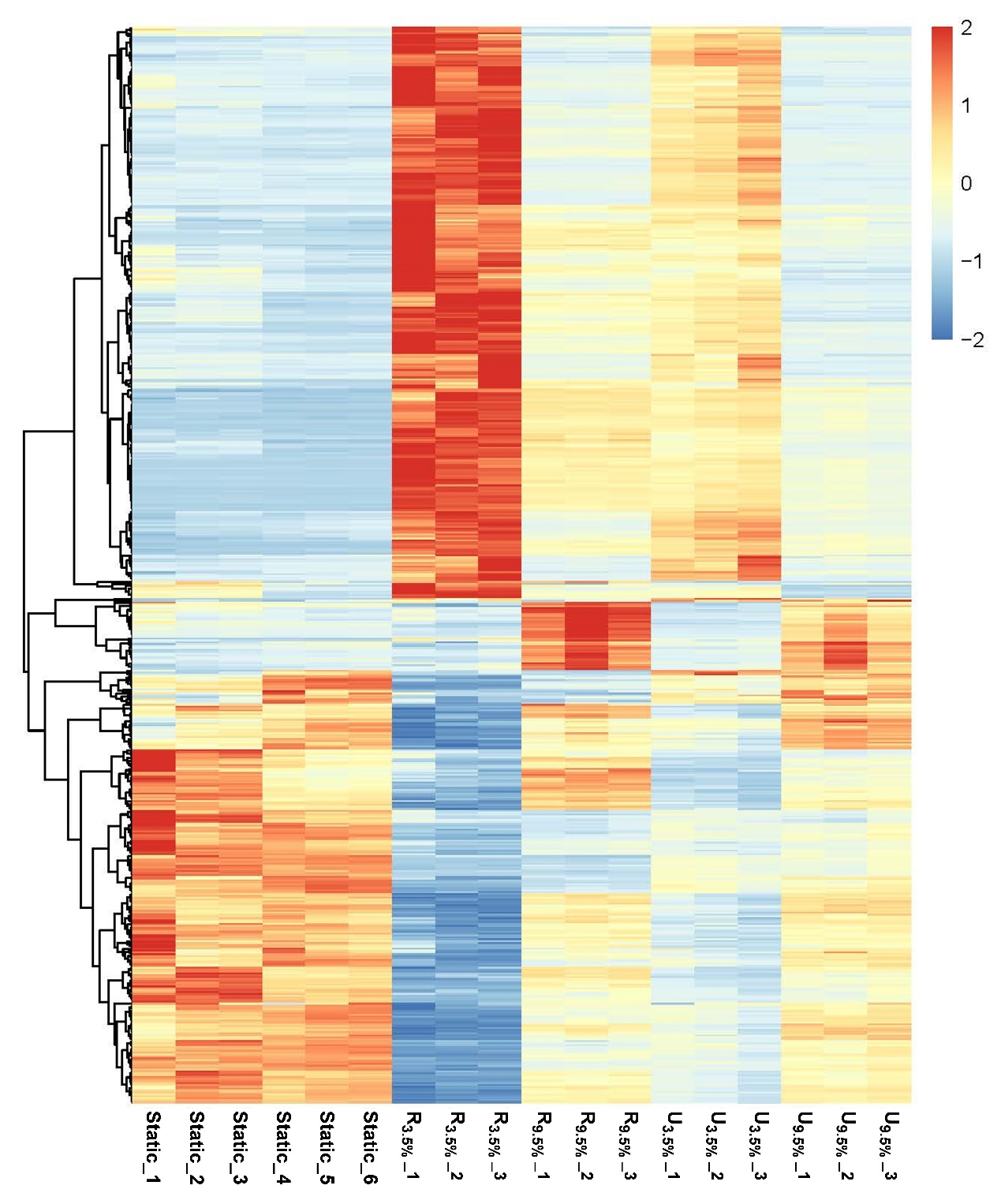


**Figure S26.** Differential expression gene clustering heatmap. This heatmap clusters all differentially expressed genes from the comparison group into sets based on similar expression patterns. Hierarchical clustering was utilised to normalized FPKM values (Z-score), with colors ranging from red (high expression) to blue (low expression), indicating normalised log2(FPKM+1) values.


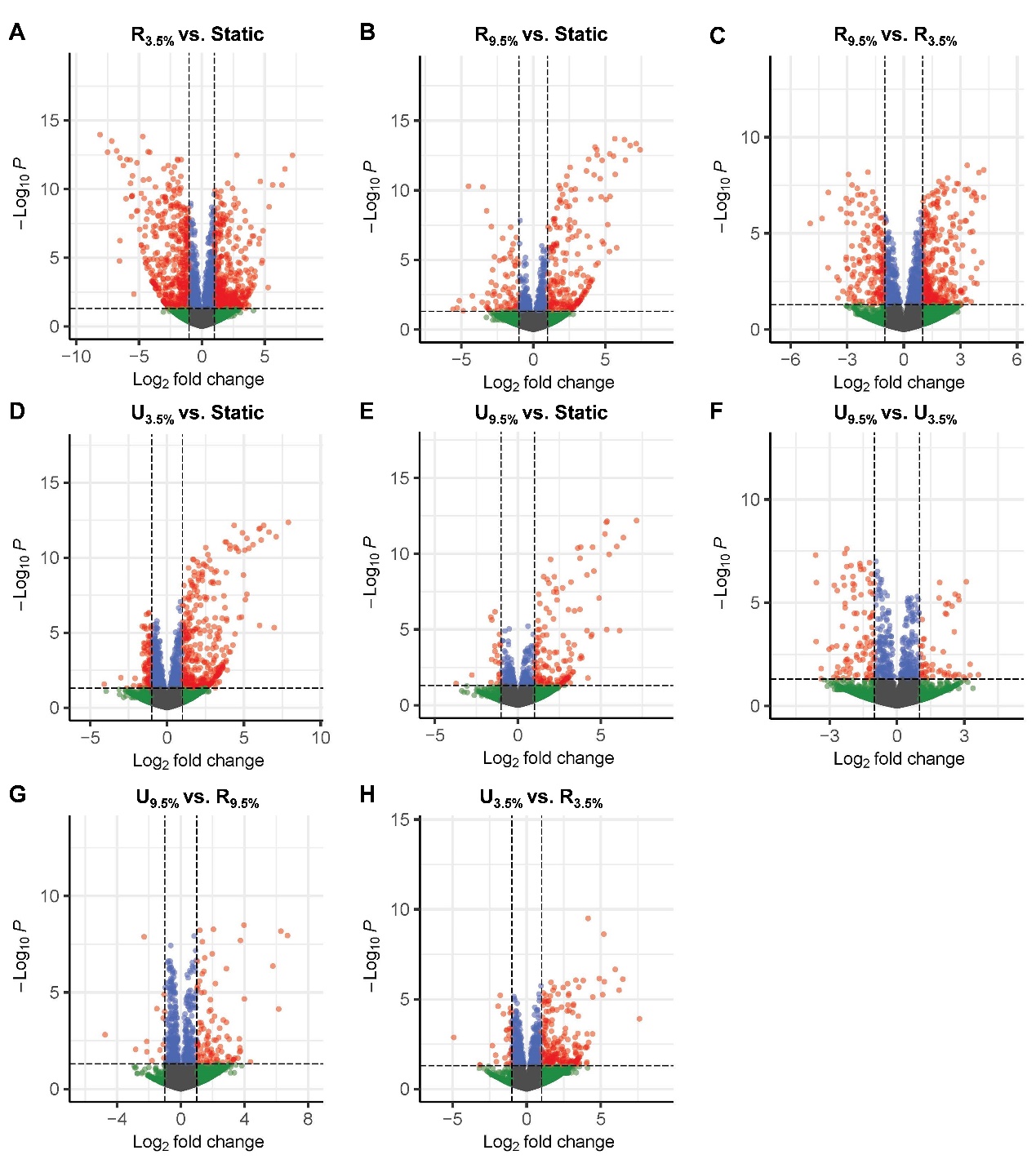
 **Figure 27.** Differential gene volcano map for RNA-seq data. A-C) Multiaxial robotic comparisons including Static vs. R_3.5%_, Static vs. R_9.5%_, R_3.5%_ vs. R_9.5%_; D-F) Uniaxial comparisons including Static vs. U_3.5%_, Static vs. U_9.5%_, U_3.5%_ vs U_9.5%_; G-H) Cross-modality comparisons of multiaxial robotic vs. uniaxial, R_3.5%_ vs. U_3.5%_, and R_9.5%_ vs. U_9.5%_. Each plot's x-axis shows the log2 fold change in gene expression, while the y-axis displays the statistical significance (-log10 padj). Red dots indicate up-regulated genes, green dots indicate down-regulated genes, and a dashed line marks the threshold for differential gene selection.


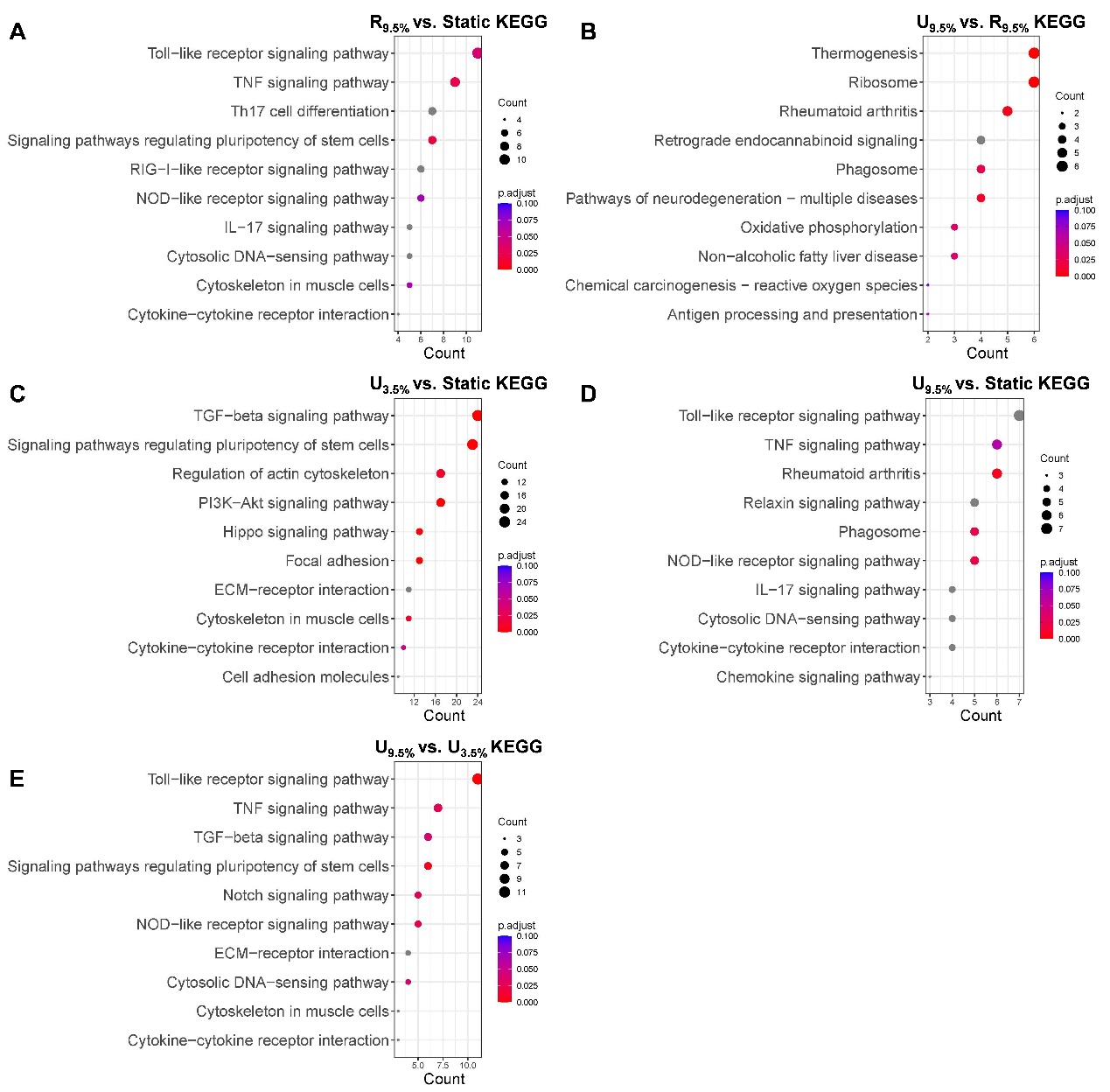


**Figure S28**. KEGG pathway enrichment for RNA-seq data. A) Multiaxial robotic: R_9.5%_ *vs.* Static; B) multiaxial robotic vs. uniaxial: R_9.5%_ vs. U_9.5%_ C-E) uniaxial: U_3.5%_ vs. Static , U_9.5%_ *vs.* Static, U_9.5%_ *vs.* U_3.5%_ . The plots show the top 10 most significant KEGG pathways related to tendon differentiation or mechanical stimulation. The x-axis represents the number of differential genes associated with each pathway. The y-axis lists the KEGG pathways. Point size indicates the number of genes annotated to each pathway, with color shading from red to purple denoting the level of significance in enrichment.


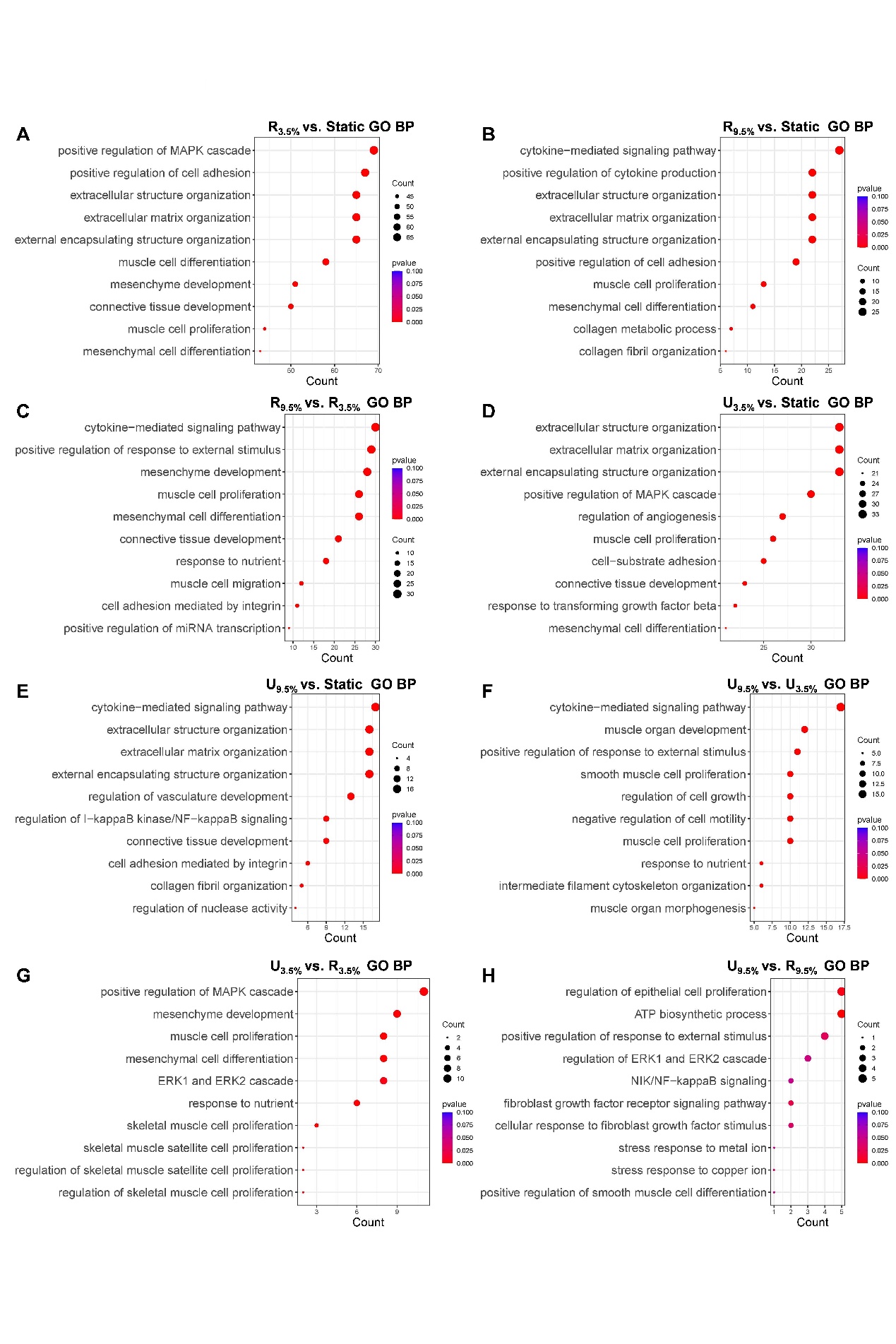


**Figure S29.** GO pathway enrichment for RNA-seq data. A-C) Multiaxial robotic: R_3.5%_ *vs.* Static, R_9.5%_ *vs.* Static, R_3.5%_ *vs.* R_9.5%_; D-F) uniaxial: U_3.5%_ *vs.* Static, U_9.5%_ *vs.* Static, U_3.5%_ *vs.* U_9.5%_; G-H) multiaxial robotic *vs.* uniaxial: R_3.5%_ *vs.* U_3.5%_, R_9.5%_ *vs.* U_9.5%_. The plots show the top 10 most significant GO pathways related to tendon differentiation or mechanical stimulation. The x-axis represents the number of differential genes associated with each pathway. The y-axis lists the GO pathways. Point size indicates the number of genes annotated to each pathway, with color shading from red to purple denoting the level of significance in enrichment.


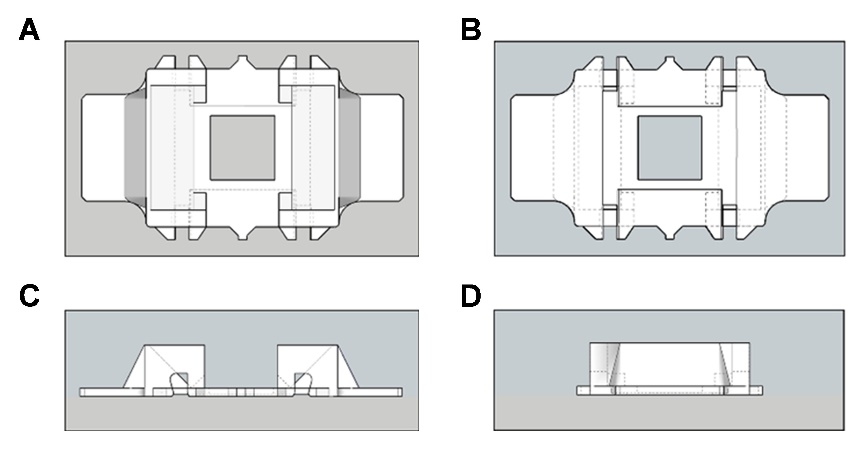


**Figure S30.** Bioreactor chamber mount views for non-invasive confocal microscopy imaging. A) Top view; B) Bottom view; C) Side view; D) Front view.

**Table S1.** RNA sequencing sample quality control

| **Sample** | **Error rate (%)** | **Q20 (%)** | **Q30 (%)** | **GC pct** |
| --- | --- | --- | --- | --- |
| Static1 | 0.03 | 97.47 | 93.41 | 53.9 |
| Static2 | 0.03 | 97.51 | 93.51 | 53.87 |
| Static3 | 0.03 | 97.67 | 93.85 | 53.77 |
| R_3.5%_ 1 | 0.03 | 97.71 | 93.85 | 53.85 |
| R_3.5%_ 2 | 0.03 | 97.66 | 93.76 | 53.43 |
| R_3.5%_ 3 | 0.03 | 97.69 | 93.82 | 53.33 |
| R_9.5%_ 1 | 0.03 | 97.56 | 93.54 | 53.82 |
| R_9.5%_ 2 | 0.03 | 97.59 | 93.59 | 53.38 |
| R_9.5%_ 3 | 0.03 | 97.59 | 93.72 | 53.82 |
| U_3.5%_ 1 | 0.03 | 96.76 | 91.94 | 51.95 |
| U_3.5%_ 2 | 0.03 | 97.13 | 92.65 | 51.83 |
| U_3.5%_ 3 | 0.03 | 96.93 | 92.38 | 52.17 |
| U_9.5%_ 1 | 0.03 | 97.19 | 92.85 | 51.74 |
| U_9.5%_ 2 | 0.03 | 96.7 | 91.84 | 51.76 |
| U_9.5%_ 3 | 0.03 | 97.1 | 92.59 | 51.69 |

Error rate: Average sequencing error rate, which is calculated by Qphred = -10log10(e).

Q20: The percentage of the bases whose Q Phred values is greater than 20. (Number of bases with Q Phred value > 20) / (Number of total bases) * 100.

Q30: The percentage of the bases whose Q Phred values is greater than 30. (Number of bases with Q Phred value > 30) / (Number of total bases) * 100.

GC pct: The percentage of G&C base numbers of total bases. (G&C base number) / (Total base number) * 100.

**Table S2.** Parameters for the Ogden model defined for the silicone core and silicone-CNT shell.

| **Parameter** | **Silicone** | **Silicone-CNT** |
| --- | --- | --- |
| $\mu_{1}$ (MPa) | 0.41 | -666.52 |
| $\mu_{2}$ (MPa) | -0.32 | 306.93 |
| $\mu_{3}$ (MPa) | 0.64 | 400.62 |
| $\alpha_{1}$ | 0.89 | -2.21 |
| $\alpha_{2}$ | 12.50 | -2.14 |
| $\alpha_{3}$ | -25.00 | -5.30 |

**Table S3.** Antibodies used in western blot.

| **Antibody** | **Source** | **Catalog. #** | **Host species** | **Diluted concentration** | **Molecular Weight (KD)** |
| --- | --- | --- | --- | --- | --- |
| p-PI3K (Tyr607) | Invitrogen | PA5-104853 | Rabbit pAb | 1:1000 | 85 |
| PI3K (p86) | Invitrogen | PA5-29220 | Rabbit pAb | 1:1000 | 85 |
| p-AKT (ser473) | Proteintech | 28731-1-AP | Rabbit pAb | 1:1000 | 58 |
| AKT | Proteintech | 10176-2-AP | Rabbit pAb | 1:1000 | 58 |
| GAPDH | Proteintech | 10494-1-AP | Rabbit pAb | 1:5000 | 36 |
| Goat anti-rabbit IgG (H+L) | Proteintech | SA00001-2 | Goat pAb | 1:5000 |  |
