# Supplementary figures and images for: Humanoid Robotic Loading Enhances Mechanotransduction in Tendon Tissue Engineering
Source: Cyborg Bionic Syst. 2026 Mar 24;7:0542. doi: 10.34133/cbsystems.0542 (PMC13009534; doi:10.34133/cbsystems.0542)

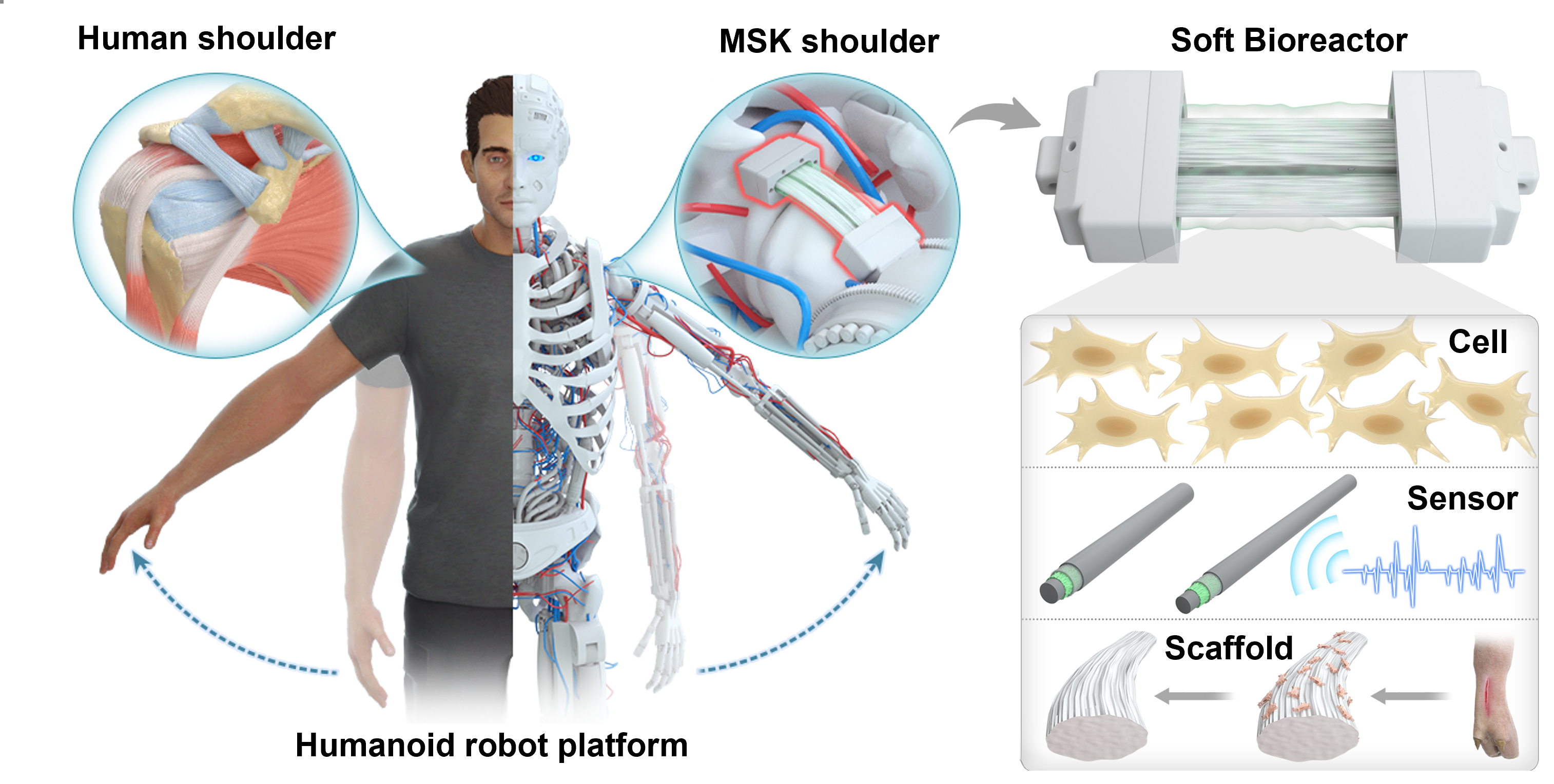

Supplement: Supplementary 1 — Graphical Abstract Figs. S1 to S30 Tables S1 to S3 [file cbsystems.0542.f1.zip › Graphical abstract.tif]
